# Engineering a production platform for the potent antipseudomonal antibiotic mureidomycin A by reprogramming the pacidamycin biosynthetic gene cluster

**DOI:** 10.1186/s12934-026-03166-9

**Published:** 2026-10-30

**Authors:** Panagiota-Hanna Koutsandrea, Richard Sonnengrün, Nils Hauck, Andreas Kulik, Theresa Harbig, Jan-Martin Daniel, Kay Katja Nieselt, Rebecca Jane Miriam Goss, Tanja Schneider, Silja Mordhorst, Harald Gross, Bertolt Gust

**Affiliations:** 1 Department of Pharmaceutical Biology, Eberhard Karls University Tübingen, Tübingen, 72076 Germany; 2 German Center for Infection Research (DZIF), Partner Site Tübingen, Tübingen, 72076 Germany; 3 Department of Microbial Bioactive Compounds, Interfaculty Institute of Microbiology and Infection Medicine, Eberhard Karls University Tübingen, Tübingen, 72076 Germany; 4 Institute for Bioinformatics and Medical Informatics, Eberhard Karls University Tübingen, Tübingen, 72076 Germany; 5 School of Chemistry, Institute of Engineering, University of St Andrews, St Andrews, KY16 9ST UK; 6 Institute for Pharmaceutical Microbiology, University of Bonn, University Hospital Bonn, Bonn, 53127 Germany; 7 German Center for Infection Research (DZIF), Partner Site Bonn-Cologne, Bonn, 53113 Germany

**Keywords:** Antipseudomonal activity, Genetic engineering, Heterologous expression, Mureidomycin, Non-ribosomal peptide synthetase, Pacidamycin, *Streptomyces albus*, Uridyl peptide antibiotics.

## Abstract

**Background:**

Uridyl peptide antibiotics display potent activity against *Pseudomonas aeruginosa*, a pathogen causing severe nosocomial infections. The four subclasses—mureidomycins, pacidamycins, napsamycins, and sansanmycins—inhibit the underexplored bacterial target phospho-MurNAc-pentapeptide translocase (MraY). Structurally, these compounds contain a 3’-deoxyuridine linked to a central diaminobutyric acid. The latter is situated within a pseudo tetra- or pentapeptide moiety, assembled by a non-ribosomal peptide synthetase machinery. The subclasses are distinguished by variations in the incorporated amino acids. Native producer strains typically synthesize numerous different uridyl peptide antibiotics in low yields. Since the pacidamycin gene cluster displays higher overall yields compared to the napsamycin cluster in identical expression systems, we aimed to reprogram it into a selective production platform for the highly bioactive mureidomycin A. This approach is intended to streamline isolation processes and support hit-to-lead optimization.

**Results:**

*Streptomyces albus* J1074 was identified as the most promising host for heterologous expression of the pacidamycin gene cluster. Subsequently, media screening and precursor feeding were conducted, and regulators of closely related gene clusters were introduced into the heterologous host. The overexpression of the NpsM regulator of the napsamycin gene cluster increased production significantly. Next, the pacidamycin gene cluster was engineered to produce the more potent mureidomycins. Variable positions of the peptide moiety were individually targeted by domain or module swaps of the non-ribosomal peptide synthetase machinery. More specifically, these were replaced by homologs of the napsamycin gene cluster, resulting in strains mainly producing sansanmycin A or mureidomycin A. Promoter engineering enhanced mureidomycin A production in the final strain, correlating with increased transcription levels in RNA sequencing experiments on an intermediate strain harboring the same promoters.

**Conclusions:**

Hybrid uridyl peptide antibiotic gene clusters were constructed by genetically engineering the pacidamycin cluster, demonstrating the versatility of these clusters. While the native pacidamycin cluster only produced pacidamycin D and S, the final strain yields the more bioactive mureidomycin A and Met-pacidamycin 5. Additionally, sansanmycin A (*syn.* Met-pacidamycin 4) can be isolated from an intermediate construct of this study. This expands the access to different analogs and highlights further engineering potential, which will facilitate the future development of these promising antibiotics.

**Graphical Abstract:**

**Figure:**
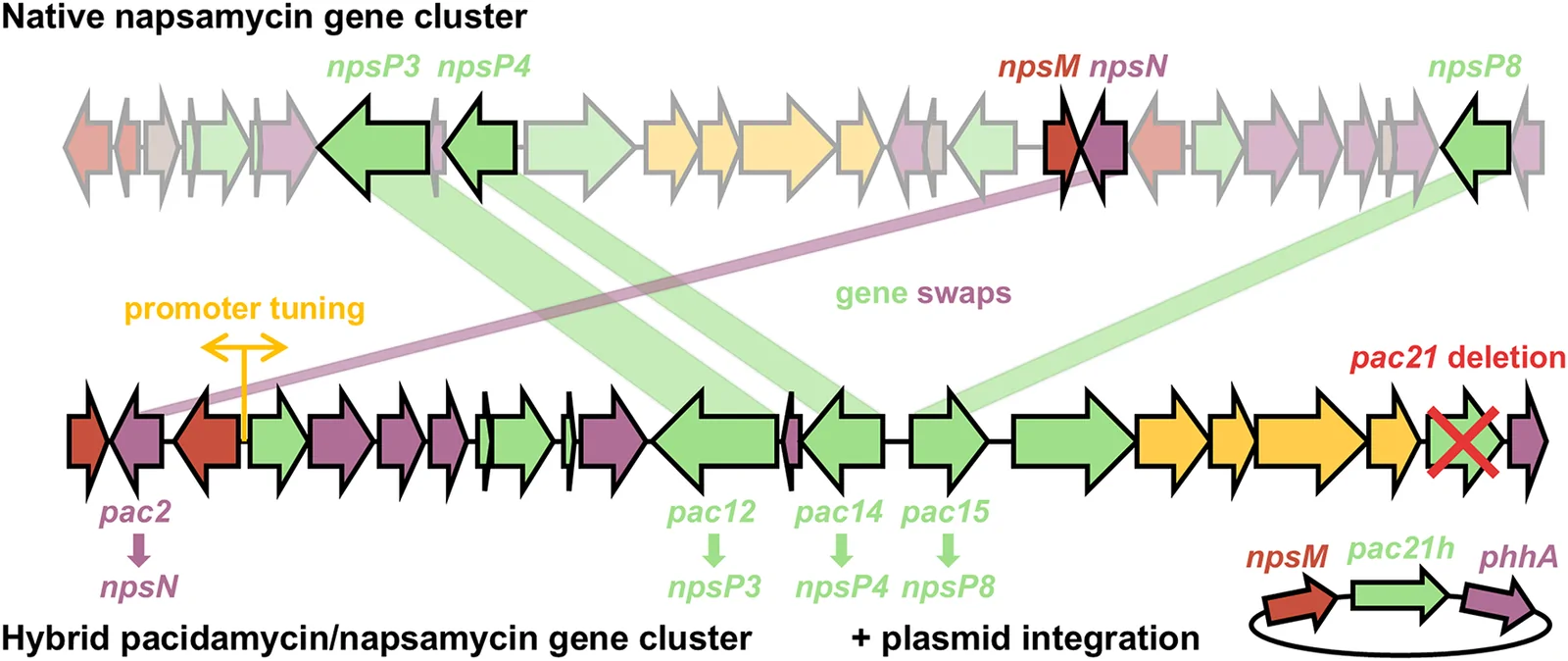

**Supplementary Information:**

The online version contains supplementary material available at https://doi.org/10.1186/s12934-026-03166-9.

## Background

The escalating levels of antimicrobial resistance against commonly administered antibiotics, noted especially in Gram-negative pathogens such as *Pseudomonas aeruginosa*, *Escherichia coli* and *Klebsiella pneumoniae*, remain a serious threat to global health [1]. This stands in sharp contrast to the few innovative antibiotics that have been approved since the year 2000, emphasizing the crucial need for research and development of novel antibacterial compounds [2], preferably agents with new chemical skeletons and novel modes of action.

**Fig. 1:**
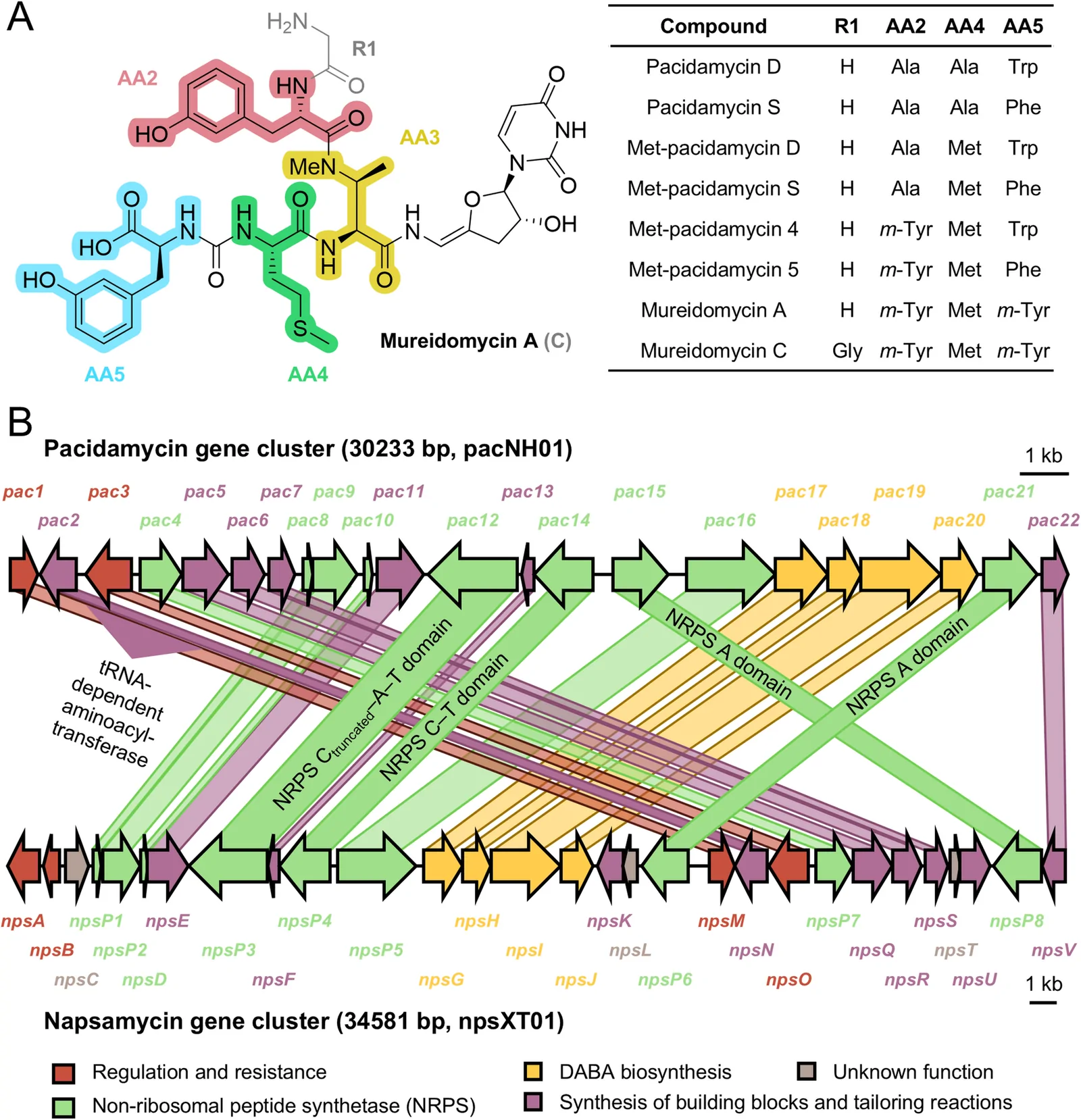
Chemical structure of mureidomycin A and C and the native pacidamycin and napsamycin biosynthetic gene clusters. (**A**) Chemical structure(s) of mureidomycin A (and C), in which the peptide backbone positions of UPAs are highlighted, as follows: position two (AA2) in red, three (AA3) in yellow, four (AA4) in green, and position five (AA5) in blue. The N-terminal amino acid (R1) of the pentapeptide mureidomycin C is shown in gray; mureidomycin A is a tetrapeptide. A table summarizing the UPAs investigated in this study and their amino acid compositions is included. Pacidamycin 4 and 5 were originally isolated by Chen et al. [4]., pacidamycin D by Fronko et al. [10]., pacidamycin S by Rackham et al. [11]., and mureidomycin A and C by Isono et al. [3]. (**B**) The native pacidamycin and napsamycin biosynthetic gene clusters are depicted. Homologous genes between the biosynthetic gene clusters are connected by colored lines; genes that are relevant for modifications of this study are labeled with the function of the respective gene products, while lines connecting genes that were not exchanged are semi-transparent

Uridyl peptide antibiotics (UPAs) are a class of natural products that exhibit potent activity against *P. aeruginosa* and thus represent promising candidates for addressing the above-mentioned unmet therapeutic need. UPAs consist of a 3’-deoxyuridine moiety, which is linked through a unique 4’,5’-enamide linkage to the core (*2 S*, *3 S*)-*N*-methyl-2,3-diaminobutyric acid moiety (DABA), located at the center of a tetra- (or penta-)pseudopeptide backbone. Interestingly, the direction of the peptide chain is reversed at the DABA moiety and at the ureido structure, situated between the two C-terminal residues of the peptide scaffold (Fig. 1A, Table S1) [3–6]. Four closely related UPA subclasses—mureidomycins, pacidamycins, napsamycins, and sansanmycins—have been reported, differing in the amino acid composition and the nucleoside core, which either consists of a 3’-deoxyuridine or a 5,6-dihydro-3’-deoxyuridine. As mentioned above, UPAs can harbor a tetra- or pentapeptide backbone. Since the N-terminal amino acid is not consistently present, residue numbering varies across the literature. For clarity and consistency, pentapeptide residues are designated as positions 1–5, whereas tetrapeptides are numbered 2–5 throughout this work. The central DABA moiety will be referred to as position three (AA3) of the UPA backbone. In natively occurring pacidamycins, l-Ala is found at position four of the scaffold (AA4), and l-Trp, l-Phe, or l-*meta*-Tyr (*m-*Tyr) at position five (AA5), while mureidomycins and napsamycins harbor l-Met at AA4 and l-*m*-Tyr at AA5. Regarding position two (AA2), l-Ala, l-*m*-Tyr, or a bicyclic moiety (Table S1) is incorporated in pacidamycins. In mureidomycins and napsamycins, only l-*m*-Tyr or a bicyclic moiety can be found at AA2. Position one of the UPA scaffold is designated “R1” and can consist of l-Ala, l-Gly, or an acetyl moiety [7]. Pacidamycins were isolated for the first time from *Streptomyces coeruleorubidus* AB 1183 F-64 [8], mureidomycins from *Streptomyces flavidovirens* SANK 60,486 [9], napsamycins from *Streptomyces* sp. HIL Y-82 [5], and sansanmycins from *Streptomyces* sp. SS [6].

UPAs display narrow-spectrum bioactivity against *P. aeruginosa* [7] and are potent inhibitors of bacterial cell wall biosynthesis by targeting the phospho-*N*-acetylmuramyl-pentapeptide translocase (also termed translocase I or MraY), which catalyzes the formation of undecaprenyl-pyrophosphoryl-*N*-acetylmuramyl-pentapeptide (lipid I) [12, 13]. The highest bioactivity against *P. aeruginosa* was reported for mureidomycins (minimal inhibitory concentrations [MICs]: 0.1–3.13 µg/mL for mureidomycin C, 0.1–25 µg/mL for mureidomycin A, 4–64 µg/mL for pacidamycins, 6.25–50 µg/mL for napsamycins, and 10–12.5 µg/mL for sansanmycins). Sansanmycins also display potent activity against *Mycobacterium tuberculosis* [5, 7, 14, 15]. Mureidomycins A and C were reported to exhibit low toxicity after intravenous injection in mice and to be effective in *P. aeruginosa* infection models in mice [14]. Thus, mureidomycin A and C are the most promising candidates for further drug development against pseudomonal infections.

The biosynthetic gene clusters (BGCs) of all four UPA subclasses have been identified. The napsamycin BGC (Fig. 1B), discovered in *Streptomyces* sp. DSM 5940, comprises 29 genes (*npsA*–*npsV*). The heterologous expression of this BGC in *Streptomyces coelicolor* M1154 led to the production of napsamycin C, as well as mureidomycin A and B [16]. The sansanmycin BGC of the original producer, *Streptomyces* sp. SS, was reported to consist of 25 genes (*ssaA*–*ssaY*) [17]. The transcriptional regulator SsaA, which is encoded within the BGC, was characterized and employed for the activation of a cryptic mureidomycin BGC in *Streptomyces roseosporus* NRRL 15998. This BGC consists of 28 open reading frames (*SSGG_02978*–*SSGG_03004*) and is highly similar to the napsamycin BGC (identities on protein level: 94–99%). This activation approach led to the production of eight distinct *N*-acetylated mureidomycins, all of them featuring a tetrapeptide backbone [18]. The pacidamycin BGC was identified in *Streptomyces coeruleorubidus* AB1183F-64 and NRRL 18370 and consists of 22 genes (*pac1*–*pac22*; Fig. 1B) [11, 19]. Pacidamycin 1–7 [4], pacidamycin 5N [11], pacidamycin D, pacidamycin 4 N, and pacidamycin 5T [10] have been isolated from the native producer strains, while pacidamycin S was reported after heterologous expression of the BGC in *Streptomyces lividans* TK24 and subsequently also detected in trace amounts in the native producer [11]. In general, the biosynthesis of UPAs is directed by a non-ribosomal peptide synthetase (NRPS) system that deviates from the typical, modular genetic organization of NRPSs, and is instead encoded by highly dissociated genes. A common biosynthetic pathway has been described for UPAs, as exemplified below by the pacidamycin BGC [20]. The central DABA moiety is biosynthesized from l-Thr and then recognized and activated by the adenylation–thiolation–thioesterase (A–T–TE) tridomain enzyme Pac16. Next, the *N*-methyltransferase Pac22 methylates DABA, and *N*-methyl-DABA is loaded onto the central T domain Pac8 by the A–T–TE tridomain Pac16. The N-terminal amino acid (AA2) or dipeptide (R1 + AA2) and the C-terminal ureido-dipeptide (AA4 + AA5) are constructed by discrete mono-, di-, or tridomain proteins and both transferred onto Pac8–*N*-methyl-DABA. Finally, the standalone condensation domain (C domain) Pac9 releases the peptide backbone from the central T domain Pac8 by catalyzing the formation of an amide linkage with 3’-deoxy-5’-aminouridine [20, 21]. UPA biosynthesis has been reviewed elsewhere in detail [7].

A key prerequisite for establishing a UPA production platform in heterologous hosts amenable to genetic manipulation is capturing the complete BGC in an appropriate vector. The pacidamycin BGC was identified and isolated using an *S. coeruleorubidus* AB1183F-64 cosmid library, resulting in a SuperCos1-derived cosmid (Stratagene), termed 2 H-5. Cosmid 2 H-5 harbors the entire pacidamycin BGC (30.3 kb). The heterologous expression of 2 H-5 in *S*. *lividans* TK24 confirmed the production of pacidamycin D and S [11, 22]. The pacidamycin BGC served as the starting point for the present study, since a preliminary heterologous expression of both, the napsamycin and the pacidamycin BGCs, resulted in higher UPA yields for the pacidamycin BGC. Besides the higher compound yield, the simpler architecture of the pacidamycin BGC is an additional benefit for conducting genetic manipulations, since it consists only of 22 genes situated on at least five operons, while the napsamycin BGC harbors a total of 29 genes situated on at least nine operons.

In this study, the heterologous expression of the pacidamycin BGC was first optimized through the systematic screening of host strains and cultivation media, regulator overexpression, and precursor feeding experiments. Next, multiple NRPS-encoding genes of the pacidamycin BGC were replaced with their homologs from the napsamycin BGC or deleted, resulting in preferential production of mureidomycin A by the engineered strain. Promoter tuning improved compound yield, which was confirmed by an RNA sequencing analysis on an intermediate strain. Overall, these efforts led to the construction of a highly engineered hybrid pacidamycin/napsamycin BGC that enables the efficient production and isolation of mureidomycin A.

## Results

### Optimized heterologous expression of the native pacidamycin BGC

**Fig. 2:**
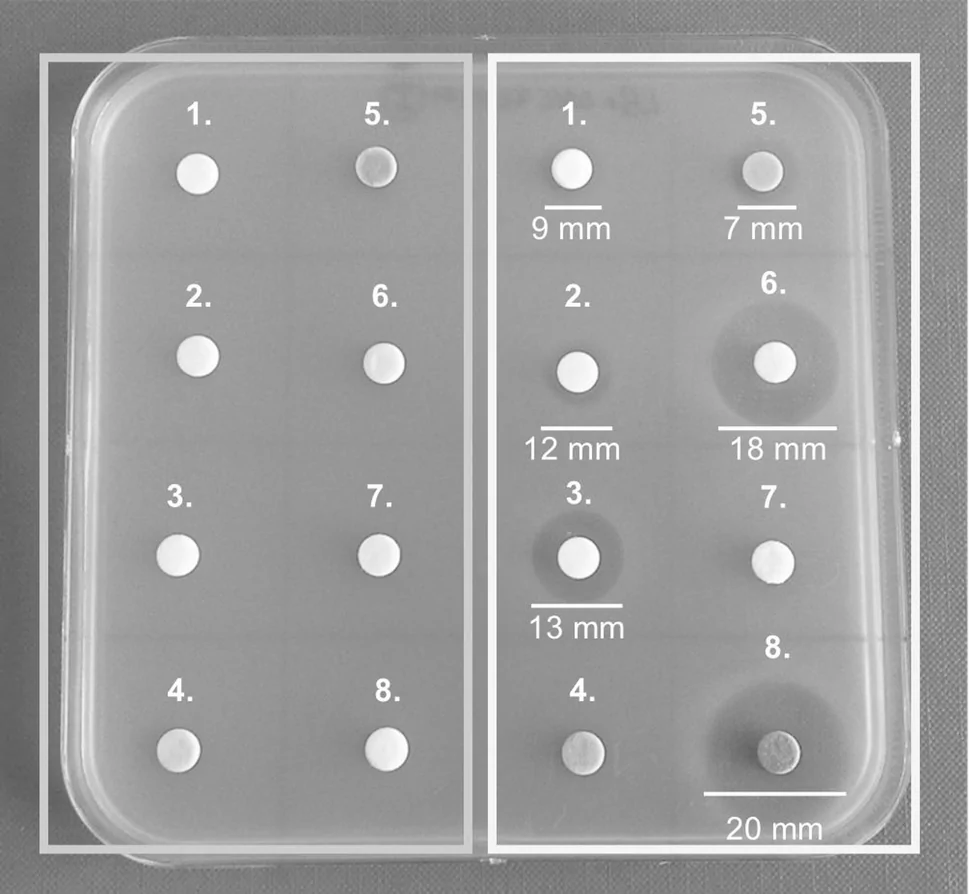
Investigation of the pacidamycin production in seven heterologous host strains in comparison with the native producer in agar diffusion assays. Crude extracts of the heterologously expressed pacidamycin BGC pacNH01 were examined in agar diffusion assays against *E. coli* BW25113Δ*tolC*. Extracts of wild-type control strains are displayed on the left, while extracts on the right side originate from the respective strains harboring pacNH01. Heterologous hosts tested in this study: (1) *S. coelicolor* M512, (2) *S. coelicolor* M1146, (3) *S. coelicolor* M1154, (4) *S. lividans* TK24, (5) *S. lividans* 1326, (6) *S. albus* J1074, (7) *S. venezuelae* ATCC 10712, 8 left) negative control (pure DMSO), 8 right) native producer *S. coeruleorubidus* NRRL 18370 (positive control)

First, the heterologous expression of the native pacidamycin BGC was established. In order to facilitate stable compound production without the need for antibiotic selection during cultivation, the pacidamycin BGC of cosmid 2 H-5 was integrated into the genome of the respective heterologous hosts. For this purpose, the ampicillin resistance marker *bla* (β-lactamase) located on the backbone of cosmid 2H-5 was replaced with an integration cassette from the plasmid pIJ787 [23]. The integration cassette consisted of the tetracycline resistance gene *tet* as a selectable marker, the origin of transfer *oriT*_RP4_ allowing efficient intergenic conjugal transfer into *Streptomyces*, and the attachment site *attP*(ϕC31) and integrase *int*(ϕC31) of phage ϕC31 for integration into the *Streptomyces* genome. The resulting integrative cosmid was termed pacNH01 (Figure S1), contained the whole native pacidamycin BGC, and was verified by sequencing. To identify the heterologous host best suited for UPA production, pacNH01 was transferred into seven widely used *Streptomyces* host strains by intergenic conjugation and successful integration into the host genome was confirmed by polymerase chain reaction (PCR) for three individual exconjugants (see Table S2 for all primers mentioned throughout this study). As preliminary screening, crude culture extracts of all obtained mutant strains were tested regarding their bioactivity in agar diffusion assays (Fig. 2). *E. coli* BW25113Δ*tolC* was used as the indicator strain, since it has been shown to be sensitive to UPAs due to a knockout of the gene encoding the efflux pump TolC [24, 25]. No zone of inhibition was evident in the seven wild-type heterologous host strains *S. coelicolor* M512, *S. coelicolor* M1146, *S. coelicolor* M1154, *S. lividans* TK24, *S. lividans* 1326, *S. albus* J1074 (now classified as *S*. *albidoflavus* J1074 [26]), and *S. venezuelae* ATCC 10,712, which were cultivated as a control without the integration of cosmid pacNH01 (Fig. 2, left side, number 1–7). In contrast, zones of inhibition were observed for five out of the seven extracts (1–3, 5, and 6). The largest zones of inhibition that indicated the highest UPA production were noted for *S. coelicolor* M1146/pacNH01, *S. coelicolor* M1154/pacNH01, and *S. albus* J1074/pacNH01 (Fig. 2, right side, number 2, 3, and 6, respectively). The latter even showed bioactivity comparable to that of the native pacidamycin producer *S. coeruleorubidus* NRRL 18370 (Fig. 2, right side, number 8).

As noted previously, the first heterologous expression of the pacidamycin BGC in *S. lividans* TK24 by Rackham et al. only yielded pacidamycin D and S. Contrastingly, the production of eleven different pacidamycins was detected in the native producer strain *S. coeruleorubidus* NRRL 18370. Pacidamycin D was produced at significantly higher levels in the heterologous host than in the native strain [11]. Out of the eleven pacidamycins produced in the native producer strain, all containing either an *m*-Tyr or a derivative thereof were not detected in the heterologous host. These are produced by a Phe-3-hydroxylase (PhhA), and the respective gene is situated elsewhere on the genome of *S. coeruleorubidus* NRRL 18370 and is therefore not included on cosmid pacNH01. Hence, only pacidamycin D and S were expected to be produced by the heterologous hosts harboring pacNH01 [11, 27, 28].

Culture extracts of the three most promising strains identified in the agar diffusion assays (*S. coelicolor* M1146/pacNH01, *S. coelicolor* M1154/pacNH01, and *S. albus* J1074/pacNH01) were analyzed by liquid chromatography coupled with mass spectrometry (LC–MS). Indeed, pacidamycin D (*m*/*z* 712 [M + H]^+^) and S (*m*/*z* 673 [M + H]^+^) were detected at the same retention times as in the native producer strain *S. coeruleorubidus* NRRL 18370. While the extracted ion chromatogram (EIC) signal intensity was increased in all three tested strains in comparison to the native producer strain, the largest increase was noted for *S. albus* J1074/pacNH01 (Figure S2), correlating with the largest zone of inhibition observed in the agar diffusion assay. Thus, this strain was chosen as the heterologous host for all subsequent experiments. It should be noted that the low LC–MS signal intensity corresponding to pacidamycin D and S in *S. coeruleorubidus* NRRL 18370 culture extracts does not contradict the bioassay data. More likely, the large zone of inhibition corresponding to the native producer can rather be attributed to the other pacidamycins that are only produced in this strain, and not the heterologous hosts.

**Fig. 3:**
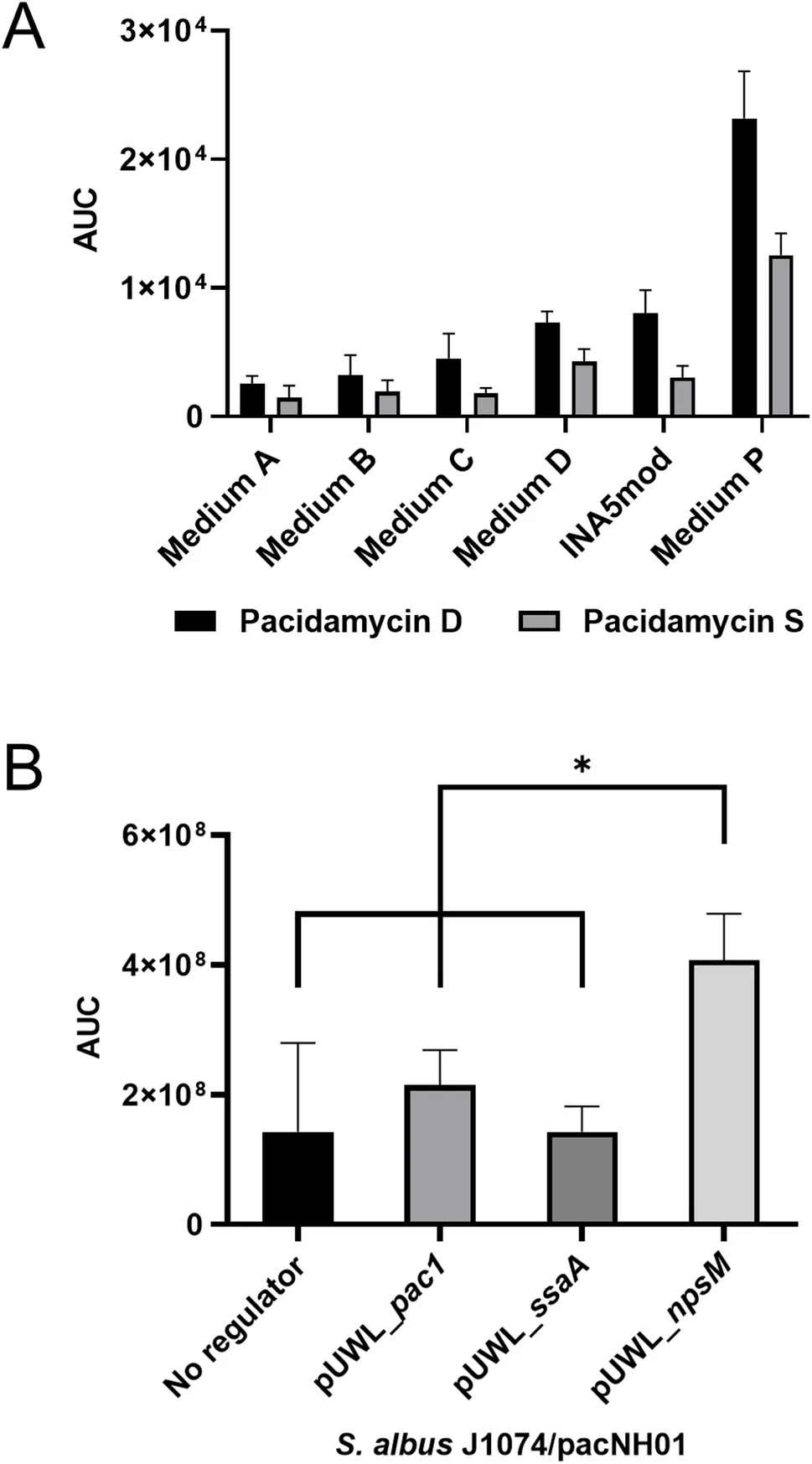
Media screening and regulator overexpression as tools for enhanced pacidamycin production. (**A**) Area under the curve (AUC) for pacidamycin D and S calculated from HPLC UV chromatograms of crude culture extracts of *S. albus* J1074/pacNH01 in six different media. (**B**) Comparison of pacidamycin D production in *S. albus* J1074/pacNH01 strains additionally harboring and overexpressing one of three regulators encoded in a UPA BGC (*pac1* from the pacidamycin BGC, *ssaA* from the sansanmycin BGC, and *npsM* from the napsamycin BGC). Statistical analysis: unpaired t-test, two-tailed P value, P value style: GraphPad, one asterisk signifies *P* ≤ 0.05, error bars indicate the standard deviation

A series of experiments was undertaken to further optimize compound yield in the heterologous host, including media screening, regulator studies, and feeding experiments. First, six different media previously utilized for UPA production were identified in the literature and tested regarding pacidamycin D and S production in *S. albus* J1074/pacNH01. For this purpose, precultures were grown in tryptic soy broth (TSB) and used to inoculate main cultures of the media A, B, C, D, P, and modified INA5 (for details, see material and methods section) in triplicates [5, 9–11, 18, 29]. After cultivation and extraction, compound production was analyzed by comparing the peak areas observed in the respective high-performance liquid chromatography (HPLC) chromatograms. Among all tested media, medium P yielded the highest levels of pacidamycin D and S (Fig. 3A). This medium has previously been utilized for the production of pacidamycins, as well as mureidomycins and napsamycins, e.g., for the identification of the napsamycin BGC [10, 16]. Thus, medium P was used for all subsequent cultivations.

Another strategy frequently employed to increase natural product biosynthesis is the overexpression of cluster-situated positive regulators. The transcriptional regulator *ssaA* located within the sansanmycin BGC was characterized as a first-in-class regulator, harboring an N-terminal fork head-associated domain and a C-terminal LuxR-type helix-turn-helix motif, and was found to be essential for sansanmycin production in *Streptomyces* sp. SS [17]. To investigate whether this positive effect on UPA production would also be exerted on pacidamycins, *ssaA* and its homologs *pac1* from the pacidamycin BGC and *npsM* from the napsamycin BGC were cloned into the replicative plasmid pUWL-*aac(3)IV*_*oriT* under the control of the strong and constitutive promoter PermE*. The resulting plasmids were verified by restriction digest analysis and sequencing and introduced into *S. albus* J1074/pacNH01 by intergenic conjugation. Extracts of cultures from verified mutant strains were applied to LC–MS analysis in triplicate. Surprisingly, the native regulator of the pacidamycin BGC Pac1 as well as SsaA did not significantly influence pacidamycin D production. Only NpsM, the regulator of the napsamycin BGC, enhanced the yield with statistical significance (Fig. 3B). This renders NpsM a promising candidate to be integrated into the final optimized production strain (see below).

Lastly, precursor feeding experiments with various amino acids were conducted, as UPAs primarily differ in their peptide sequence. Pacidamycins, unlike napsamycins or mureidomycins, harbor an l-Ala at position four of the UPA scaffold. Moreover, pacidamycin D and S are identical except for position five of the scaffold, which consists of an l-Trp or l-Phe, respectively. While feeding 0.5 mM–2 mM l-Ala to main cultures of *S. albus* J1074/pacNH01 slightly increased pacidamycin D and S yields (up to ~ 50% increase; see Table S3), feeding of l-Trp or l-Phe at 0.5 mM–10 mM had no observable effect on the yield of either compound (data not shown). Since *m*-Tyr-containing UPAs are not produced in the heterologous host *S. albus* J1074, feeding this amino acid was also tested. Pacidamycin D and S do not contain *m*-Tyr and were found to be decreased 4- to 5-fold in concentration when this amino acid was added to the cultures (Table S3). Simultaneously, one new peak was detected in the HPLC chromatogram at an earlier retention time. LC–MS analysis suggests this to be pacidamycin T, which differs from pacidamycin D and S by harboring an *m*-Tyr at the C-terminus (AA5; data not shown), as previously described by Grüschow et al. [27]. None of the other *m*-Tyr-containing UPAs were detected.

### Integration of l-Met at position four of the UPA scaffold

After establishing a heterologous production platform for pacidamycins and enhancing the compound yield, steps were taken to direct the biosynthesis towards analogous compounds with greater bioactivity, i.e. the mureidomycins. This is facilitated by the unique structure of the biosynthetic machinery synthesizing UPAs. In most cases, an NRPS constitutes a linear peptide assembly line of large multi-domain modular architecture, in which subunits interact by specific docking domains [30–32]. However, in the case of UPAs, the NRPS domains and modules are of a highly dissociated nature with atypical domain–domain interactions. Multiple freestanding single, di- or tridomain proteins contribute to the biosynthesis, while the respective genes are distributed over the entire BGCs [11, 16, 19, 20]. The high degree of fragmentation of these NRPS-encoding genes allows for the exchange of single domains without disrupting unintended parts of the machinery. Although the genetic organization of UPA BGCs differs, the napsamycin cluster contains homologs of all pacidamycin genes (Fig. 1B). This enables the targeted replacement of individual domain-encoding genes of the pacidamycin BGC by their napsamycin counterparts, thereby modulating the spectrum of produced UPAs. A distinct difference between pacidamycins and mureidomycins is the presence of an l-Ala or l-Met at position four of the amino acid scaffold, respectively. Therefore, this moiety was selected as the initial target to be modified. The amino acid at position four is incorporated into UPAs *via* an A domain, which recognizes and activates a specific amino acid, l-Ala in the case of Pac15 from the pacidamycin BGC and l-Met in the case of NpsP8 from the napsamycin cluster. Their loading partners Pac14 and NpsP4 are comprised of a C–T didomain, to which the respective amino acid of Pac15 or NpsP8 is tethered before being linked to the central DABA moiety. Thus, two consecutive gene swaps were designed to exchange *pac15* for *npsP8* and *pac14* for *npsP4* on cosmid pacNH01, to allow for the incorporation of l-Met at position four of the scaffold. All genetic modifications conducted in this study, as well as the resulting BGCs, bacterial strains, and their major products, are summarized in Table 1.

**Table 1:** Overview of the sequential engineering steps, the resulting BGCs and bacterial strains, and the major products at each stage. A detailed graphical overview of the BGCs is displayed in Figure S1

Step	Genetic modification	Resulting BGC	Plasmid integration	Resulting strain	Major products
0	None, native BGC	pacNH01	None	*S*. *albus* J1074/ pacNH01	Pacidamycin D, pacidamycin S
1	*pac15* ◊ *npsP8* *pac14* ◊ *npsP4*	pacNH01 _*npsP8P4*	None	*S*. *albus* J1074/ pacNH01_*npsP8P4*	Met-pacidamycin D, Met-pacidamycin S
2	*pac2* ◊ *npsN*	pacRS01	None	*S*. *albus* J1074/ pacRS01	Met-pacidamycin D, Met-pacidamycin S
–*	Bidirectional promoter	pacHK03	None	*S*. *albus* J1074/ pacHK03	Met-pacidamycin D, Met-pacidamycin S
3	Δ*pac21*	pacRS02	pRS02 (*pac21h*, *phhA*)	*S*. *albus* J1074/ pacRS02/pRS02	Met-pacidamycin 4, Met-pacidamycin 5, mureidomycin A
			pRS04 (*pac21h*, *phhA*, *npsM*)	*S*. *albus* J1074/ pacRS02/pRS04	
4	*pac12* ◊ *npsP3*	pacRS03	pRS04	*S*. *albus* J1074/ pacRS03/pRS04	Mureidomycin A, Met-pacidamycin 5
5	Bidirectional promoter	pacHK11	pRS04	*S*. *albus* J1074/ pacHK11/pRS04	Mureidomycin A, Met-pacidamycin 5

^*^ This modification was conducted on pacRS01 but not further pursued at this time. Instead, step 3 was carried out on pacRS01, excluding cosmid pacHK03 from the engineering sequence

**Fig. 4:**
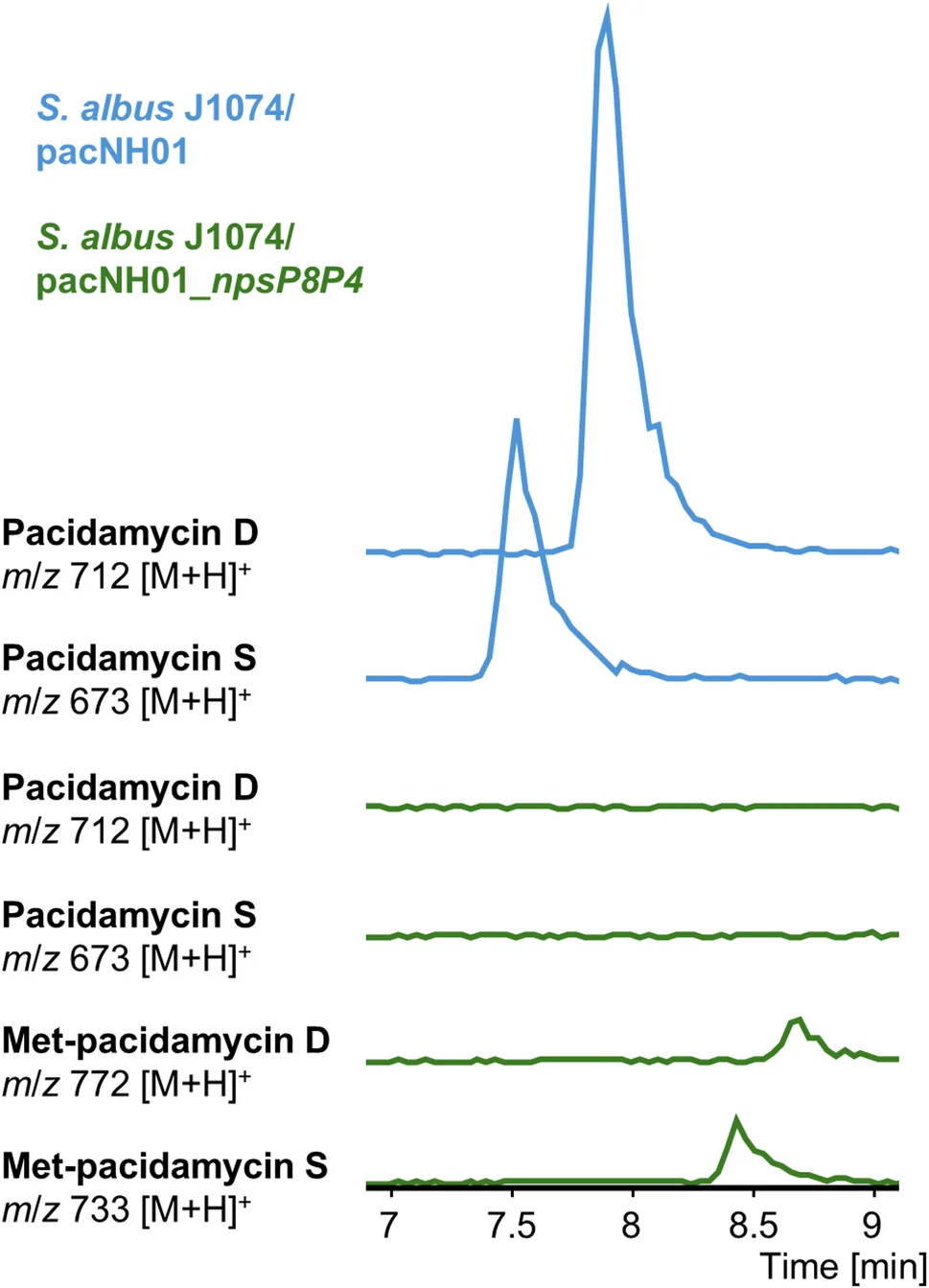
Comparison of UPA production before and after *pac15*/*npsP8* and *pac14*/*npsP4* swaps by LC–MS analysis. Extracted ion chromatograms (EICs) of LC–MS runs performed in positive mode on crude extracts of *S. albus* J1074/pacNH01 (blue) extracted for pacidamycin D (*m*/*z* 712 [M + H]^+^) and pacidamycin S (*m*/*z* 673 [M + H]^+^), and *S. albus* J1074/pacNH01_*npsP8P4* (green) extracted for pacidamycin D (*m*/*z* 712 [M + H]^+^), pacidamycin S (*m*/*z* 673 [M + H]^+^), Met-pacidamycin D (*m*/*z* 772 [M + H]^+^), and Met-pacidamycin S (*m*/*z* 733 [M + H]^+^). The chromatograms were neither offset for visualization nor normalized. Figure S5 displays the EICs including the y-axis (intensity), which is omitted here for simplification of the stacked traces

For this purpose, a cassette containing an apramycin resistance gene and the rare recognition sequence of the restriction enzyme I-SceI was constructed and integrated into pacNH01, resulting in the deletion of the target gene after Red/ET-mediated homologous recombination [33]. The obtained intermediate cosmid was subsequently linearized with I-SceI. Red/ET-proficient *E. coli* cells were then co-transformed with the linear cosmid and a linear gene fragment, containing the target gene and overhangs homologous to the cosmid. This allowed for re-circularization *via* homologous recombination, which was sufficient as the only selection mechanism. Following this method, first *pac15* was knocked out and replaced seamlessly by *npsP8*, and then the procedure was repeated for *pac14* and *npsP4*, resulting in cosmid pacNH01_*npsP8P4* (Figure S1). The cosmid was verified by enzymatic restriction analysis and sequencing prior to intergenic conjugation with *S. albus* J1074. Individual exconjugants were verified by colony PCR and cultivated in triplicate. LC–MS analysis of the crude extracts of the generated mutant strains revealed that production of pacidamycin D and S was abolished, while two new peaks corresponding to masses *m*/*z* 772 [M + H]^+^ and *m*/*z* 733 [M + H]^+^ appeared at a later retention time (Fig. 4). The observed mass shift in comparison to pacidamycin D and S corresponds to the integration of l-Met instead of l-Ala into the UPA structure. Furthermore, an LC–MS/MS analysis confirmed these new molecules to be novel analogs of pacidamycin D and S (Figure S3, S4), which harbor l-Met instead of l-Ala at position four of the UPA scaffold. These compounds were termed Met-pacidamycin D (*m*/*z* 772 [M + H]^+^) and Met-pacidamycin S (*m*/*z* 733 [M + H]^+^). It should be noted that the tentative structural assignment of these novel derivatives is based solely on LC–MS/MS data and will require confirmation by high–resolution MS (HRMS) and/or nuclear magnetic resonance (NMR) spectroscopy in future studies, once sufficient material is available. Since no UPAs harboring an l-Ala at position four could be detected in the modified strain, the NRPS engineering can be considered successful, although the production levels of the new derivatives were significantly lower than those of pacidamycin D and S in *S. albus* J1074/pacNH01.

### Attempts to integrate l-Gly at position one of the UPA scaffold and investigation of transcription levels

**Fig. 5:**
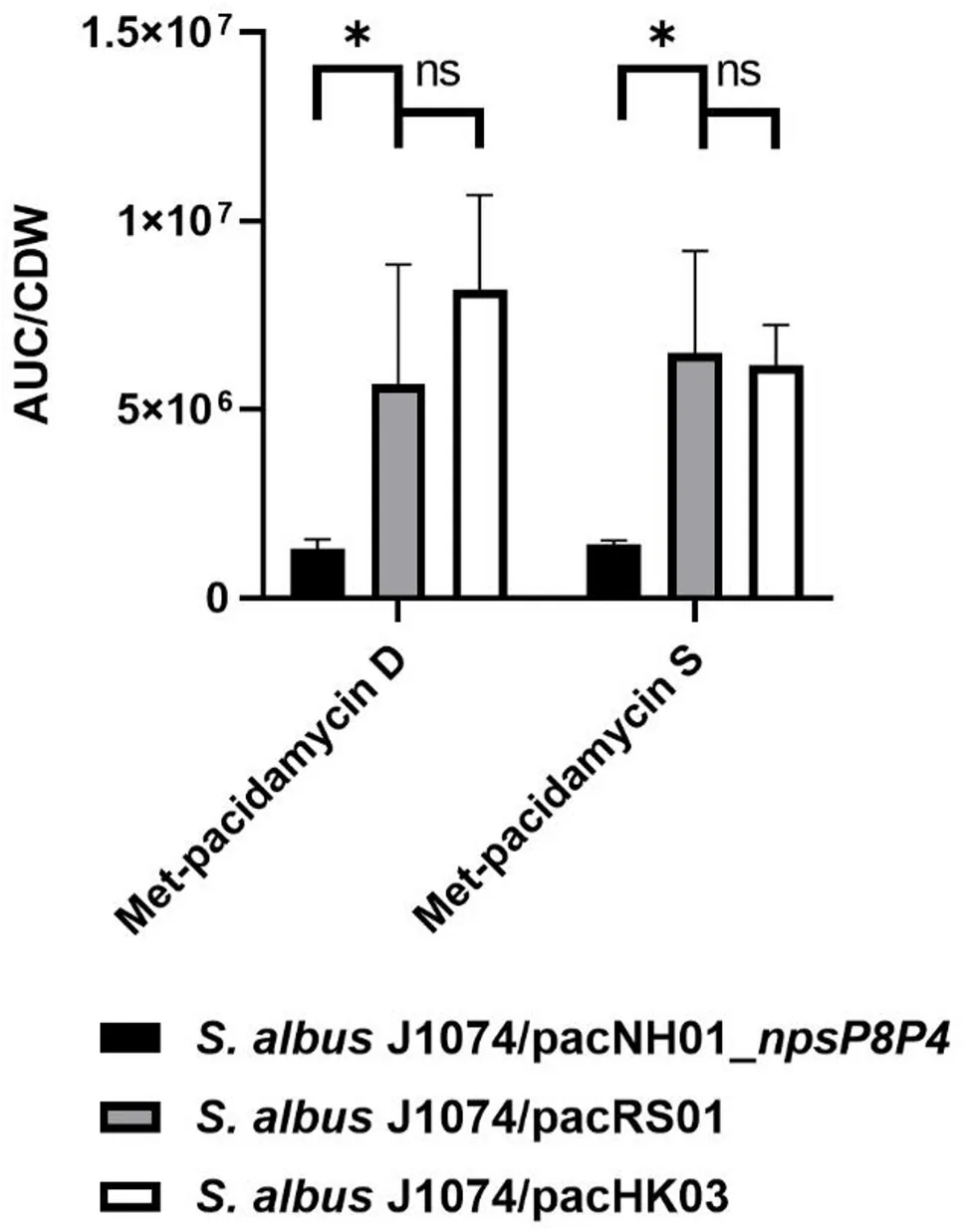
Influence of the replacement of the tRNA-dependent aminoacyltransferase-encoding gene *pac2* and the bidirectional promoter on UPA production. LC–MS analysis of Met-pacidamycin D (*m*/*z* 772 [M + H]^+^) and Met-pacidamycin S (*m*/*z* 733 [M + H]^+^) production in *S. albus* J1074/pacNH01_*npsP8P4* (black), *S. albus* J1074/pacRS01 (gray), and *S. albus* J1074/pacHK03 (white) culture extracts. AUCs of the respective EICs were determined in triplicate and normalized by cell dry weight (CDW). Statistical analysis: unpaired t-test, two-tailed P value, P value style: GraphPad, one asterisk signifies *P* ≤ 0.05; ns = not significant, error bars indicate the standard deviation

Thus far, all UPAs produced by the pacidamycin BGC *via* heterologous expression were tetrapeptides. Pentapeptide UPAs that harbor an additional l-Ala or l-Gly at the N-terminus (R1) have been described in literature [3, 4, 14]. Mureidomycins with an l-Gly at R1 display favorable bioactivity in contrast to their tetrapeptide analogs [14]. Interestingly, this additional amino acid is not incorporated by the NRPS machinery, but by the tRNA-dependent aminoacyltransferase Pac2 of the pacidamycin BGC. Pac2 catalyzes the incorporation of l-Ala, while its homolog NpsN encoded in the napsamycin BGC is selective for l-Gly incorporation [34]. Following the previously described methodology for gene swaps, *pac2* was replaced by *npsN* on pacNH01_*npsP8P4*, yielding cosmid pacRS01 (Table 1, Figure S1). Unfortunately, no pentapeptide UPA was detected in any of the culture extracts of three independent mutants of *S. albus* J1074/pacRS01, even when the medium was supplemented with additional l-Gly (data not shown). Notably, Met-pacidamycin D and S were detected in culture extracts at higher concentrations than in the parental strain *S. albus* J1074/pacNH01_*npsP8P4* (Fig. 5).

A possible explanation for the absence of pentapeptide UPAs is an insufficient transcription of *npsN*. This limitation can be addressed by replacing a native promoter upstream of *npsN* with a strong, constitutive, and synthetic promoter. Rather than introducing a unidirectional promoter directly upstream of *npsN*, we chose to insert a strong bidirectional promoter cassette between *pac3* and *pac4*, which are divergently oriented. This strategy also offers the advantage of simultaneously enhancing the transcription of *pac3*, which encodes a major facilitator transporter, and of one of the largest operons in the BGC, spanning *pac4* to *pac11*. This could potentially improve overall compound production. The overexpression of the cluster-situated transporter might additionally function as an enhanced self-resistance mechanism, since UPAs are actively exported out of the producer strain. The selected bidirectional promoter cassette consisted of promoters P15 and P16 (Table S4), as previously reported by Montiel et al. [35]. Between the promoters, an apramycin resistance gene is located and is flanked by flippase recognition target (FRT) sites to later remove the antibiotic resistance gene through a FLP recombinase-mediated reaction. The cassette was knocked in between *pac3* and *pac4* by Red/ET-mediated homologous recombination, thus replacing the native promoters. Thereby, P15 was inserted upstream of *pac3* and P16 upstream of *pac4*. The apramycin resistance was removed *via* a FLP recombinase-mediated reaction, resulting in cosmid pacHK03 (Table 1), which was verified by enzymatic restriction analysis and sequencing. The obtained mutant strain *S. albus* J1074/pacHK03 was verified by PCR analysis and cultivated in triplicate alongside *S. albus* J1074/pacRS01 and *S. albus* J1074/pacNH01_*npsP8P4* as references. The following LC–MS analysis of culture extracts revealed a slight, but not statistically relevant increase in compound production for Met-pacidamycin D. The relatively high variability observed in the production of this compound by *S. albus* J1074/pacRS01 and *S. albus* J1074/pacHK03 may have limited the ability to detect statistically significant differences between the strains. Thus, a positive effect of the promoter on compound formation cannot be fully excluded. Met-pacidamycin S production was unaffected and once again no pentapeptide UPAs could be detected (Fig. 5).

In order to investigate the enhanced compound production caused by replacing *pac2* with *npsN* and the lesser effect of the bidirectional promoter cassette, the total mRNA of strains containing the native pacidamycin BGC (pacNH01), the mutated BGC with all three gene swaps (pacRS01), and one additionally harboring the bidirectional promoter cassette (pacHK03) was isolated, reverse transcribed into cDNA, and sequenced. As expected, transcription rates were considerably reduced by the gene swaps conducted on pacNH01 to obtain pacRS01, while an insertion of the bidirectional promoter cassette resulting in pacHK03 strongly increased the amount of transcripts found for the entire BGC and not only for the operons, which were directly affected by the exchanged promoters (Figures S6-S9). Since this does not translate into compound yield, there might be a different, hitherto unidentified, bottleneck.

It should be noted that the comparison of transcripts of pacRS01 vs. pacHK03 was conducted in a different strain, *S. albus* J1074/pRS02, which additionally harbors plasmid pRS02 (discussed in the following chapter), on which genes for the integration of *m*-Tyr at position two of the UPA scaffold are encoded. However, the only difference between the two strains *S. albus* J1074/pacRS01/pRS02 and *S. albus* J1074/pacHK03/pRS02 was the integration of the bidirectional promoter cassette, which led to a drastic increase in the transcription of biosynthetic genes. Moreover, transcription rates in *S. albus* J1074/pacRS01 and *S. albus* J1074/pacRS01/pRS02 were almost identical regarding the pacidamycin BGC (Figures S7 and S8) and, thus, the integration of plasmid pRS02 into the genome does not seem to affect BGC transcription. It can therefore be assumed that valid conclusions can be drawn by comparing these strains.

It could be hypothesized that the enhanced UPA yield in *S. albus* J1074/pacRS01 arises from the knockout of *pac2*, which catalyzes the transfer of an alanyl residue from alanyl-tRNA and might therefore contribute to depleting the l-Ala pool available for Met-pacidamycin D and S biosynthesis. However, at this point, this is purely speculative, and further experiments will need to be conducted to form a proper hypothesis. Although no Gly-containing UPAs were detected in any culture extracts, we decided to carry out further modifications on pacRS01 instead of returning to pacNH01_*npsP8P4*, since pacRS01 harbors the potential for facilitating l-Gly incorporation in the future and exhibits an improved compound production. Since the integration of the bidirectional promoter resulted in elevated transcription levels, it will be reintroduced into the cosmid after all gene swaps are completed. This will allow the effects of further modifications on compound production to be assessed based solely on engineering of the NRPS machinery, uncoupled from the influence of synthetic promoters.

### Integration of l-*m*-Tyr at position two of the UPA scaffold and the influence of a positive regulator on production

In the pacidamycin scaffold, l-Ala or l-*m*-Tyr occupy position two (AA2). In contrast, mureidomycins solely harbor l-*m*-Tyr or bicyclic derivatives thereof at this position. Interestingly, the heterologous expression of the pacidamycin BGC yielded only UPAs containing l-Ala at AA2. Thus, investigations into the origin of l-*m*-Tyr were prompted, which concluded that this non-canonical amino acid is synthesized from Phe by the Phe-3-hydroxylase PhhA. The respective gene *phhA* was identified in the genome of the native producer *S. coeruleorubidus* NRRL 18370 adjacent to *pac21h*. On the amino acid level, *pac21h* displays 90% sequence similarity and 84% sequence identity to the NRPS A domain Pac21, encoded in the pacidamycin BGC. While Pac21 incorporates l-Ala, its ortholog Pac21h activates and integrates l-*m*-Tyr into the UPA scaffold at AA2. Since neither *phhA* nor *pac21h* are situated within the pacidamycin BGC, UPAs harboring l-*m*-Tyr are not synthesized in the heterologous host [27].

**Fig. 6:**
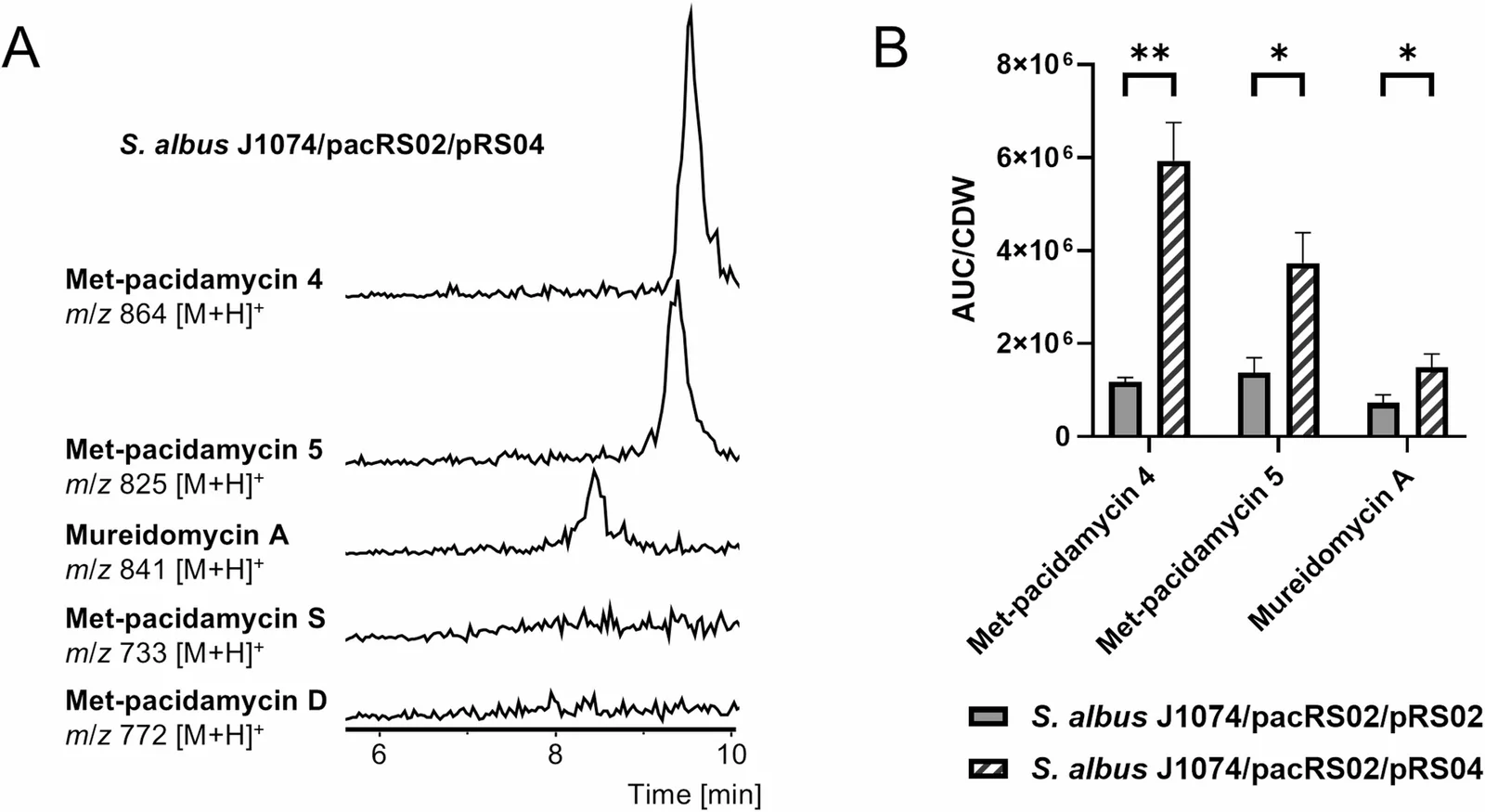
UPA production in *S. albus* J1074/pacRS02/pRS04 and investigation of the influence of NpsM on compound yield. (**A**) Extracted ion chromatograms (EICs) of LC–MS analysis performed in the positive mode of *S. albus* J1074/pacRS02/pRS04 culture extracts. Top to bottom: extracted for Met-pacidamycin 4 (*m/z* 864.3 [M + H]^+^), Met-pacidamycin 5 (*m/z* 825.3 [M + H]^+^), mureidomycin A (*m/z* 841.3 [M + H]^+^), Met-pacidamycin S (*m/z* 733.2 [M + H]^+^), and Met-pacidamycin D (*m/z* 772.3 [M + H]^+^). (**B**) LC–MS analysis of culture extracts of *S. albus* J1074/pacRS02/pRS02 (gray) and *S. albus* J1074/pacRS02/pRS04 (striped). AUCs of the respective EICs were determined in duplicate or triplicate and normalized by cell dry weight (CDW). Statistical analysis: unpaired t-test, two-tailed P value, P value style: GraphPad, one asterisk signifies *P* ≤ 0.05, two asterisks signify *P* ≤ 0.01, error bars indicate the standard deviation. The chromatograms were neither normalized nor horizontally offset for visualization. Figure S14 shows the EICs of panel A with the y-axis (intensity), which has been omitted here to simplify the stacked traces

In order to direct the biosynthesis towards mureidomycins, *pac21* was deleted on cosmid pacRS01 by Red/ET-mediated homologous recombination utilizing the apramycin cassette of plasmid pIJ773, which was subsequently removed by a FLP recombinase-mediated reaction. The resulting cosmid pacRS02 (Table 1, Figure S1) was conjugated with *S. albus* J1074, and culture extracts of this mutant strain were investigated regarding their metabolite profile. Indeed, UPA production was abolished, since no A domain capable of incorporating amino acids at position two was present in the strain (Figure S10A). Next, the knockout was complemented with *pac21h*. For this purpose, the integrative plasmid pMVM3100 was chosen as the vector, due to its apramycin resistance and its integration at the alternative attachment site *attB*(ϕBT1) [36], which does not compete with the *attP*(ϕC31)-containing cosmids utilized herein. Firstly, *pac21h* and *phhA* were cloned into pMVM3100, thus producing plasmid pRS02. The integrative plasmid posed an opportunity for the incorporation of the positive regulator NpsM of the napsamycin BGC, which had previously enhanced UPA production. Thus, plasmid pRS02 was further modified to include *npsM* under the control of the strong constitutive promoter PermE*, yielding pRS04. Both plasmids were verified by enzymatic restriction analysis and sequencing and were subsequently conjugated into *S. albus* J1074/pacRS02. UPA production was restored in culture extracts of the verified mutant strains *S. albus* J1074/pacRS02/pRS02 and *S. albus* J1074/pacRS02/pRS04 (Table 1; Figs. 6A and S10B). As expected, production of Met-pacidamycin D and S was no longer detected. Instead, their counterparts, which differ only in the incorporation of l-*m*-Tyr at position two, were readily detected and verified by LC–MS/MS analysis and termed Met-pacidamycin 4 (*m/z* 864.3 [M + H]^+^) and Met-pacidamycin 5 (*m/z* 825.3 [M + H]^+^), respectively (Figures S11 and S12). As noted above for Met-pacidamycin D and S, the assignments of Met-pacidamycin 4 and 5 are likewise based on LC–MS/MS data and will require confirmation by HRMS and/or NMR spectroscopy in future studies. In the literature, the compound herein referred to as Met-pacidamycin 4 is also named sansanmycin A [17]. Moreover, the target compound mureidomycin A (*m/z* 841.3 [M + H]^+^) was also detected in the extracts in lesser amounts (Figs. 6A and S13). The positive regulator of the napsamycin BGC once again exerted beneficial effects on compound production, since the integration of pRS04 increased production of all three produced UPAs significantly, when compared to strains harboring solely plasmid pRS02 (Fig. 6B).

### Integration of l-*m*-Tyr at position five of the UPA scaffold and promoter engineering to enhance yield

**Fig. 7:**
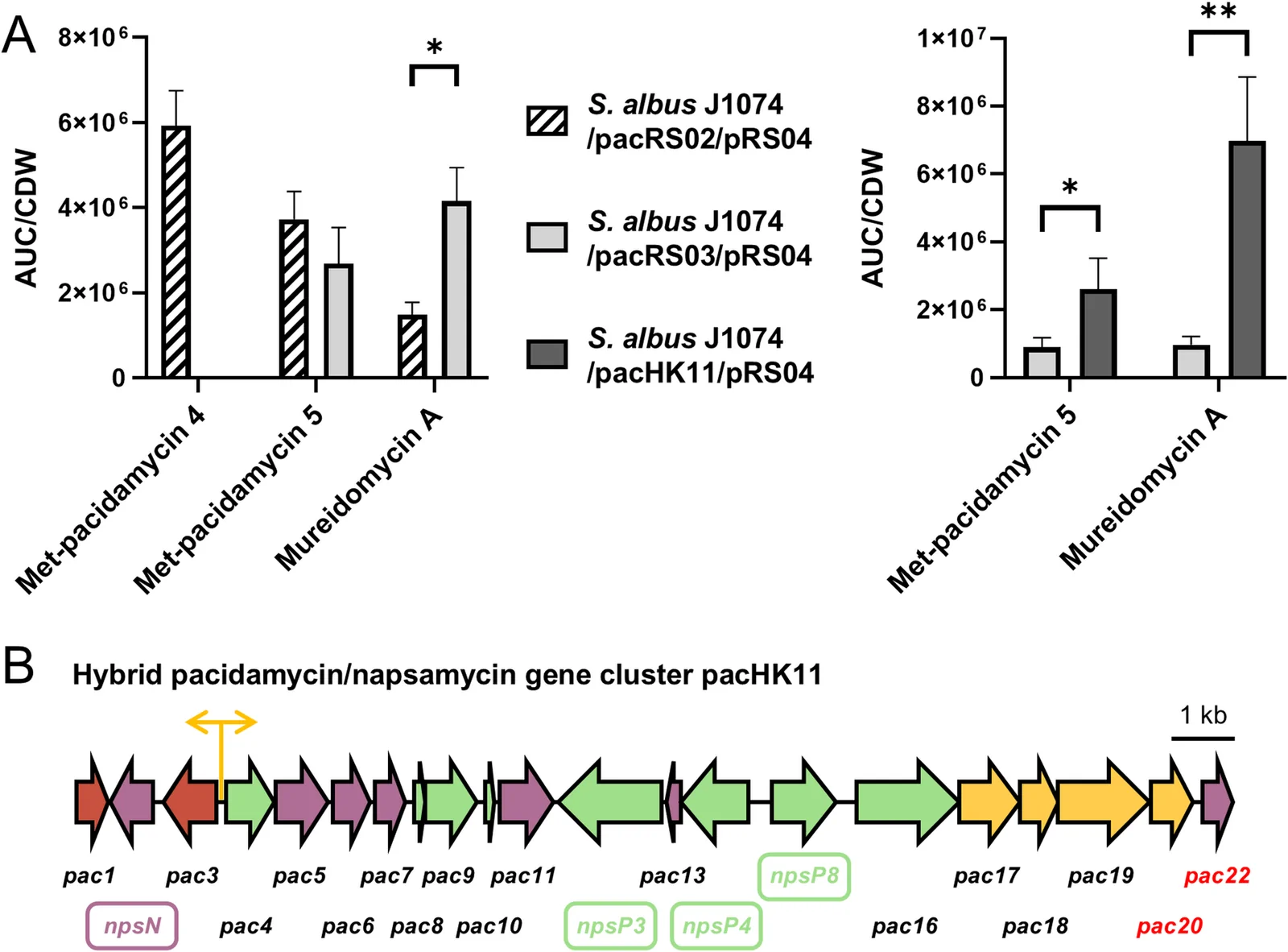
UPA production in *S. albus* J1074/pacRS02/pRS04 prior and subsequent to replacing *pac12* with *npsP3* and investigation of the influence of the bidirectional promoter cassette. (**A**) Compound yield determined by LC–MS analysis of culture extracts. Left side: *S. albus* J1074/pacRS02/pRS04 (striped) and *S. albus* J1074/pacRS03/pRS04 (light gray). Right side: *S. albus* J1074/pacRS03/pRS04 (light gray) and *S. albus* J1074/pacHK11/pRS04 (dark gray). AUCs of all respective EICs were determined in duplicate or triplicate and normalized by cell dry weight (CDW). Statistical analysis: unpaired t-test, two-tailed P value, P value style: GraphPad, one asterisk signifies *P* ≤ 0.05, two asterisks signify *P* ≤ 0.01, error bars indicate the standard deviation. (**B**) Genetic organization of the final BGC of this study, the hybrid pacidamycin/napsamycin gene cluster pacHK11. Genes that were exchanged with napsamycin gene cluster homologs are marked with a box around the respective name. The synthetic bidirectional promoter is marked with yellow arrows above the gene cluster. Names of genes flanking the knockout site are marked red

The last variable position of the peptide moiety of UPAs is the C-terminus or position five (AA5). The three UPAs produced by the hitherto constructed strain *S. albus* J1074/pacRS02/pRS04 are Met-pacidamycin 4, Met-pacidamycin 5, and mureidomycin A, which only differ in the C-terminal position by harboring l-Trp, l-Phe, or l-*m*-Tyr, respectively. In the pacidamycin BGC, *pac12* encodes a C_trunc_–A–T domain (C_trunc_: truncated C domain), and the A domain recognizes and activates AA5. Out of all three UPAs produced by *S. albus* J1074/pacRS02/pRS04, Met-pacidamycin 4 displayed the highest signal intensity, possibly indicating a preference of Pac12 for l-Trp, followed by l-Phe (present in Met-pacidamycin 5). Only traces of mureidomycin A, which harbors l-*m*-Tyr at AA5, were detected. Contrastingly, the homologous A–T domain of the napsamycin BGC, NpsP3, strongly favors l-*m*-Tyr, as evidenced by the fact that this amino acid forms the C-terminus in all described mureidomycins and napsamycins. Thus, *pac12* was substituted by *npsP3* on cosmid pacRS02, following the aforementioned domain swap technique, yielding cosmid pacRS03 (Table 1, Figure S1). Culture extracts of the resulting mutant strains *S*. *albus* J1074/pacRS03/pRS04 were analyzed as described above. Solely Met-pacidamycin 5 and mureidomycin A production was detected, while Met-pacidamycin 4 synthesis had been abolished. In contrast to the progenitor strain, the highest signal intensity was detected for mureidomycin A, which harbors l-*m*-Tyr at position five. AA5 of Met-pacidamycin 5 (l-Phe) can likely be incorporated due to the close structural similarity with l-*m*-Tyr, while l-Trp (AA5 of Met-pacidamycin 4) is not accepted by NpsP3 (Fig. 7A and S15). The hybrid pacidamycin/napsamycin BGC produces the target compound of this study, mureidomycin A, as the major product, and the dehydroxylated variant Met-pacidamycin 5 is produced as a minor compound. In the final step of genetic engineering, promoter tuning was performed to improve the compound yield. For this purpose, the previously described bidirectional promoter cassette was integrated between *pac3* and *pac4* of pacRS03, resulting in cosmid pacHK11 (Table 1, Figure S1). Increased transcription levels had previously been observed in the mRNA sequencing data for the same promoter exchange on cosmid pacRS01. As expected based on these data, a significant enhancement of mureidomycin A production was observed (~ 4-fold). Thus, the pacidamycin BGC was reprogrammed in the present work by NRPS engineering from a cluster solely producing pacidamycins to the final strain *S. albus* J1074/pacHK11/pRS04, which mainly produces mureidomycin A and Met-pacidamycin 5 (Fig. 7B and S16).

Since *S. albus* J1074 displayed the highest UPA production of the tested streptomycetes, its successor strain *S. albus* B2P1 was employed to test if compound production could be further enhanced. This strain had been engineered by deleting 15 specialized metabolite gene clusters from the genome and then integrating an additional *attB*(ϕC31) site into the genome of *S. albus* J1074, which previously harbored two *attB*(ϕC31) sites. These modifications had reduced the complexity of the metabolic background and increased compound production during test expression of various natural products [37, 38]. Therefore, the final cosmid pacHK11 and plasmid pRS04 were integrated into *S. albus* B2P1. The resulting strain *S. albus* B2P1/pacHK11/pRS04 was verified by colony PCR and cultivated simultaneously with *S. albus* J1074/pacHK11/pRS04 as the reference strain to investigate the effect on compound production. The following LC–MS analysis of crude extracts revealed Met-pacidamycin 5 production to be increased ~ 6.8-fold and mureidomycin A production to be increased ~ 5.5-fold in comparison to the reference strain, supporting a beneficial effect of increased cosmid copy number on compound production (Figure S16).

### Inhibitory activity of investigated UPAs

Finally, the inhibitory activity of the UPAs against their target enzyme MraY was assessed using purified MraY translocase from *P. aeruginosa*. The lowest IC₅₀ value was determined for Met-pacidamycin 4 (sansanmycin A) at 0.0016 µg/mL (0.0019 µM), followed by mureidomycin A at 0.0033 µg/mL (0.0039 µM), pacidamycin D at 0.0029 µg/mL (0.0041 µM), and pacidamycin S at 0.0064 µg/mL (0.0095 µM) (Figure S18). The UPAs were subsequently evaluated in vivo against *P. aeruginosa* PAO1, displaying MIC values ranging from 1 to 8 µg/mL. Consistent with the biochemical inhibition data, Met-pacidamycin 4 exhibited the lowest MIC (1 µg/mL), followed by mureidomycin A (2 µg/mL), whereas pacidamycin D and pacidamycin S showed higher MIC values (8 µg/mL). Collectively, these findings indicate that, among the UPAs investigated, the presence of l-Met at position AA4 is associated with greater antibacterial activity than l-Ala. This observation suggests that mureidomycin-type compounds may represent more promising candidates for further development as anti-infective agents against *P. aeruginosa* than the corresponding pacidamycins.

## Discussion

The UPAs mureidomycin A and C comprise intriguing candidates for hit-to-lead compound development in drug discovery, since they inhibit the unexploited protein MraY that is not targeted by any antibiotic on the market [39]. Besides the initially reported cytoplasmic binding site, some UPAs also bind to MraY at a second periplasmic inhibition site, which is a crucial benefit—reducing the development of resistance against UPAs [13]. Recent structure–activity relationship studies of UPAs reasoned that their amino acid composition greatly influences antibacterial activity. Hydrophobic residues in position four (AA4) of the scaffold improve cellular uptake, l-*m*-Tyr at the N-terminus of a tetrapeptide backbone (AA2, as in mureidomycin A) increases MraY inhibition, and the ureido-dipeptide motif (AA4 + AA5) is crucial for MraY inhibition. Additionally, it was shown that the NppA1A2BCD transporter, which belongs to the nucleoside peptide permease (Npp) family and, thus, transports peptides, is responsible for the uptake of 3’-hydroxymureidomycin A into *P. aeruginosa* PAO1. This compound is a synthetic derivative of mureidomycin A that differs only in the hydroxylation of the ribose moiety. Thus, the peptide backbone of UPAs might influence import selectivity [40]. All of the above underscore the importance of the NRPS engineering system of the present work, since numerous strains that produce UPAs of different amino acid combinations can be constructed and the resulting compounds can then be investigated regarding bioactivity and their effect on cell uptake and resistance.

In a previous study of our laboratory, the napsamycin BGC was identified in *Streptomyces* sp. DSM 5940 and shown to produce napsamycin C, mureidomycin A, and mureidomycin B as major compounds [16]. To establish a production platform for mureidomycin A, we first expressed the same napsamycin BGC in *S. albus* J1074, but the compound yield was low (~ 13 mg/L for mureidomycin A and B combined; data not shown). Contrastingly, the heterologous expression of the native pacidamycin BGC of pacNH01 in *S. albus* J1074 led to yields of ~ 60 mg/L and ~ 35 mg/L of pacidamycin D and S, respectively. Thus, we attempted to genetically engineer the pacidamycin BGC to facilitate the production of the closely related mureidomycins. Reprogramming the pacidamycin BGC necessitated NRPS domain swaps to allow for the incorporation of different amino acids into the scaffold that are not present in pacidamycins. As these modifications were expected to lower UPA yield, different methods to enhance compound production and optimize heterologous expression conditions were implemented first.

One strategy to improve compound production is precursor feeding, in this case the supplementation of amino acids. While feeding l-Ala to cultures of strains harboring the pacidamycin BGC improved pacidamycin yield, supplementing l-Phe or l-Trp did not influence production. Strains with the napsamycin BGC were fed with l-Gly or l-Met, which are part of the mureidomycin scaffold, but no production improvement was detected. Interestingly, feeding l-*m*-Tyr to the strain harboring the pacidamycin BGC led to the production of pacidamycin T, which only differs from pacidamycin D and S in the C-terminal l-*m*-Tyr. At this stage, however, the observed yield of ~ 30 mg/L was too low to be considered a viable alternative to genetic modifications, especially when factoring in the additional costs associated with l-*m*-Tyr feeding.

Another potential bottleneck was postulated to be the transcription of the pacidamycin BGC. To address this, several bidirectional promoter cassettes were constructed and knocked in between *pac3* and *pac4*, as well as between *npsP4* and *npsP8*, but only the insertion of the promoter cassette consisting of promoters P15 and P16 increased UPA yield (data for other cassettes not shown). Transcription of the pacidamycin BGC in *S. albus* J1074/pacNH01 (native pacidamycin BGC), *S. albus* J1074/pacRS01 (additionally harbors the gene swaps for positions one and four of the UPA scaffold), *S. albus* J1074/pacRS01/pRS02, and *S. albus* J107/pacHK03/pRS02 (additionally harbors promoter cassette P15–P16) was investigated by sequencing the total mRNA of these strains. This revealed that the gene swaps had drastically decreased cluster transcription rates in comparison to the native BGC, while the insertion of promoter cassette P15–P16 was found to increase transcription to levels higher than detected for the native cluster. Another noteworthy point revealed in the mRNA sequencing data was the low transcription of the operon encoding the proteins responsible for DABA biosynthesis. Future experiments to increase DABA supply and thus eliminate this potential bottleneck are planned but lie beyond the scope of the present work.

A feature that was described to be beneficial for the bioactivity of mureidomycins is the presence of an N-terminal l-Gly within the peptide scaffold [14]. The respective derivative of mureidomycin A that only differs in the additional l-Gly (R1) is mureidomycin C, which displays enhanced bioactivity against *P*. *aeruginosa* (MIC < 0.1–3.13 µg/mL for different strains) [14]. As mentioned above, the tRNA-dependent aminoacyltransferase of the pacidamycin BGC (Pac2), which incorporates l-Ala at R1, was replaced with the napsamycin BGC homolog NpsN (67% identity), which incorporates l-Gly [34]. However, no Gly-containing pentapeptide UPAs were detected in the resulting strains. In a study on the sansanmycin BGC, analogs with an additional N-terminal l-Gly were detected at maximal yield on day three of fermentation [41]. However, when cultures of mutant strains of this study were extracted at earlier timepoints, production of mureidomycin C was again not detected (data not shown). A possible explanation was identified by the abovementioned RNA sequencing experiments, which showed *npsN* to be hardly transcribed prior to the insertion of promoter cassette P15–P16. A unidirectional constitutive promoter was inserted upstream of *npsN* to test whether transcription is the limiting factor for l-Gly incorporation, but once again no pentapeptide-containing UPAs were detected in the culture broth (data not shown). A separate study to facilitate the biotechnological production of mureidomycin C with this platform is ongoing, while semi-synthetic approaches are also being considered. However, this is beyond the scope of the present work.

The reprogramming of the pacidamycin BGC towards a cluster that yields mureidomycin A as the major product is facilitated by the highly dissociated nature of key biosynthetic genes, which are the NRPS domains and modules. Numerous examples of successful engineering have been reported for canonical NRPSs (e.g., studies on the odilorhabdin BGC [42]). To the best of our knowledge, the engineering of a highly dissociated NRPS UPA gene cluster is demonstrated for the first time in the present study. Moreover, it represents a proof of concept that shows how different UPAs can be produced from related BGCs *via* NRPS engineering. Further engineering steps that steer the platform towards different UPAs can be envisaged and would enable the production of numerous UPAs for further preclinical testing.

## Conclusions

UPAs are a group of promising antibacterial agents that display potent bioactivity especially against the nosocomial pathogen *Pseudomonas aeruginosa*. These molecules differ in specific positions of the scaffold, by which four subclasses can be distinguished. While pacidamycins have been found to be less active, mureidomycins, and especially mureidomycin A and C, display potent bioactivity against a panel of *P. aeruginosa* strains [7, 14]. In the present work, we created a production platform that enables the selective production mureidomycin A as the major compound, and only one dehydroxylated analog as a minor product. Also, an intermediate strain produces Met-pacidamycin 4 (also known as sansanmycin A) as the major compound and can be used as a source for this interesting antibiotic with bioactivity against *Mycobacterium tuberculosis*. This was achieved by genetically engineering the pacidamycin BGC towards mureidomycin production. To the best of our knowledge, this represents the first time that a hybrid UPA BGC was constructed. More specifically, the previously produced pacidamycins were modified in all three variable positions of the UPA scaffold (AA2, AA4, and AA5), and promoter engineering to improve compound yield was also achieved. Thus, the biosynthesis was successfully directed towards the desired compounds with improved bioactivity. In contrast to the native mureidomycin/napsamycin producer *Streptomyces* sp. DSM5940, mureidomycin A is the major compound in the engineered strain. While the most bioactive derivative, mureidomycin C, was not produced by the engineered strain, mureidomycin A only differs in the absence of an l-Gly at the N-terminus (R1). It is therefore an ideal candidate for semisynthetic approaches to obtain mureidomycin C. At the same time, the variety of mureidomycins and napsamycins, which is produced in the native strain, is not present herein, greatly facilitating compound isolation and purification. This paves the way for further research on this promising compound class, e.g., by semisynthetic derivatization, which could contribute to the further development process potentially leading to preclinical studies.

## Materials and methods

### Bacterial strains, culture conditions, plasmids, cosmids, reagents, and oligonucleotides

All routine cloning procedures were carried out in the host *Escherichia coli* XL1-Blue (Stratagene). Intergenic conjugation was achieved *via E. coli* ET12567 [43]. These strains were cultivated at 37 °C on LB agar plates or in LB medium under agitation at 200 rpm. Additionally, *E. coli* BW25113/pIJ790 (for Red/ET-mediated recombination) and *E. coli* BT340 (for resistance marker removal by FLP-recombination) were cultivated, as previously reported [33, 44, 45]. *Streptomyces* strains were grown and maintained on mannitol soya flour (MS) agar plates supplemented with MgCl_2_ (1 mg/mL) at 30 °C [46]. If required, the following antibiotics were added to *E. coli* and *Streptomyces* cultures: kanamycin (50 µg/mL), apramycin (50 µg/mL), carbenicillin (100 µg/mL), chloramphenicol (25 µg/mL), and nalidixic acid (25 µg/mL). The following media were used for main cultures: medium A (3% glucose, 0.5% starch, 0.6% peptone, 0.7% (NH_4_)_2_SO_4_, 0.2% CaCO_3_), medium B (4 g/L yeast extract, 10 g/L malt extract, 4 g/L glucose), medium C (2% soluble starch, 1.5% glucose, 0.3% soytone, 0.3% peptone, 0.2% NaCl, 0.2% corn steep liquor, 0.2% CaCO_3_, and 0.2% (NH_4_)_2_SO_4_, Desmophen antifoam, adjusted to pH 6.0), medium D (3% glucose, 1% pressed yeast, 3% soybean meal, 0.4% CaCO_3_, 0.2% MgSO_4_ × 7 H_2_O, and 0.01% Nissan Disfoam CB-442), medium INA5mod (15 g/L soya flour, 5 g/L CaCO_3_, 30 mL/L glycerol, 2 g/L NaCl, adjusted to pH 7.3), and medium P (production medium; 10 g/L soytone, 10 g/L soluble starch, 20 g/L d-maltose, supplemented with 150 µL trace elements solution: 40 mg/L ZnCl_2_, 200 mg/L FeCl_3_ × 6 H_2_O, 10 mg/L CuCl_2_ × 2 H_2_O, 10 mg/L MnCl_2_ × 4 H_2_O, 10 mg/L Na_2_B_4_O_6_ × 10 H_2_O, and 10 mg/L (NH_4_)_6_Mo_7_O_24_ × 4 H_2_O) [5, 9–11, 18, 29]. Media components and chemicals were obtained from standard commercial sources. Molecular biological reagents (e.g., enzymes) were purchased from New England Biolabs (NEB, Ipswich, MA, USA), while PCR purification and gel extraction kits were purchased from Qiagen (Hilden, Germany). All bacterial strains, plasmids, and cosmids of this study are described in Table S5. The oligonucleotides designed for this work were purchased from Eurofins Genomics (Ebersberg, Germany, Table S2).

### DNA isolation and manipulation

DNA isolation and manipulation was achieved by following standard procedures published for *E. coli* and *Streptomyces* [46, 47]. DNA sequencing was conducted at Eurofins Genomics (Ebersberg, Germany).

### Construction of plasmids for regulator overexpression and integrative plasmids pRS02 and pRS04

The genes *ssaA*, *pac1*, and *npsM* encoding the regulators of the sansanmycin, pacidamycin, and napsamycin BGCs, respectively, were ordered from Integrated DNA Technologies (Newark, NJ, USA) as gBlocks™ with overhangs harboring EcoRV and SpeI restriction sites (Table S6). After enzymatic restriction of the vector pUWL-*aac(3)IV*_*oriT* and the gBlocks with EcoRV and SpeI, the genes were individually cloned into the vector by ligation with T4 DNA ligase (NEB, Ipswich, MA, USA). In the generated constructs, the genes encoding UPA regulators were situated under the control of the strong constitutive promoter PermE*. The three resulting plasmids pUWL-*aac(3)IV*_*oriT_ssaA*, pUWL-*aac(3)IV*_*oriT_pac1*, and pUWL-*aac(3)IV*_*oriT_npsM* were verified by enzymatic restriction analysis and sequencing. Intergenic conjugation with *S. albus* J1074/pacNH01 was achieved by transferring the plasmids into the methylation-deficient *E. coli* ET12567/pUZ8002, which functioned as a shuttle. The presence of the plasmid was verified in three individual exconjugants by colony PCR with primers pUWL_F and pUWL_R.

For the construction of the plasmids pRS02 and pRS04, the integrative plasmid pMVM3100 was kindly provided by Dr. Marta Vaz Mendes (University of Porto, Portugal). Plasmid pSG123 was utilized as a template for *pac21h* and *phhA* [27]. The two genes were amplified by PCR with primers pMVM3100_*pac21h*_F and pMVM3100_*pac21h*_R, digested and ligated into the SpeI/HindIII restriction sites of pMVM3100, thus producing plasmid pRS02. Subsequently, *npsM* was amplified by PCR out of the previously described pUWL-*aac(3)IV*_*oriT_npsM* alongside its promoter PermE* with primers pUWL-ermE+*npsM*_F and pUWL-ermE+*npsM*_R. The obtained fragment was then digested and ligated into the SpeI restriction site of plasmid pRS02, resulting in pRS04. Both plasmids were verified by sequencing and conjugated as described above.

### Generation of *pac21* knockout mutant

The knockout of *pac21* was achieved as previously reported [33], utilizing the resistance cassette of plasmid pIJ773, which is widely used for gene deletion through Red/ET-mediated homologous recombination. In this plasmid, the apramycin resistance gene is flanked by FRT sites, to allow for a subsequent FLP recombinase-mediated marker removal. The entire *aac(3)IV-oriT* cassette was excised in an enzymatic restriction of pIJ773 with HindIII/EcoRI and amplified by PCR with primers *PacU*_KO_F and *PacU*_KO_R, which harbor a 39-nt sequence homologous to the flanking regions of the target gene. In parallel, *E. coli* BW25113/pIJ790 was transformed with pacRS01, and the resulting strain was used for Red/ET-mediated recombination with the purified PCR product. After selection with apramycin and cosmid isolation, the apramycin resistance was removed *via* FLP-mediated recombination. The final cosmid pacRS02 was verified by restriction digest analysis and sequencing of the region of interest.

### Promoter replacements

A bidirectional promoter cassette was knocked into the pacidamycin BGCs of pacRS01 and pacRS03, thus replacing the native promoters previously situated at this site. This cassette is derived from the apramycin resistance cassette of plasmid pIJ773 [33], to which promoters P15 and P16 [35] are fused on either side of the FRT sites flanking the apramycin resistance gene. The entire cassette was amplified by PCR with primers BidPrCas1_FW_vol2 and BidPrCas1_RV, which contain the necessary overhangs complementary to the 5’-ends of *pac3* and *pac4*, where the cassette was to be inserted. Thus, P15 was inserted upstream of *pac3* and P16 upstream of *pac4*. The resulting PCR products were purified and then used for Red/ET-mediated homologous recombination, as described above.

For the construction of the cosmid pacHK11, a modified strategy was employed, since pacRS03 already contained an FRT site, rendering the use of FLP recombinase unsuitable. Thus, the PCR product used for Red/ET-mediated recombination was amplified from pacHK03 with primers PC1_on_RS03_Amp_FW and PC1_on_RS03_Amp_RV, thereby introducing long overhangs to the fragment. These overhangs were complementary to the 5’-ends of *pac3* and *pac4.* Simultaneously, the cosmid was prepared as noted above, but a different apramycin cassette (*aac(3)IV*-MRS-ISceI) was knocked in between *pac3* and *pac4*. This cassette, which was ordered as a gBlock (Table S6) and cloned into pBluescript II SK(-), additionally contained the rare restriction site for the enzyme I-SceI, which was amplified with primers ISceI_pacRS03_PS1_FW and ISceI_pacRS03_PS1_RV and facilitated the subsequent linearization of the cosmid. The following Red/ET-mediated recombination was conducted by adding the linearized cosmid to *E. coli* BW25113/pIJ790 at the same time as the purified PCR product. Verification of both constructs was achieved by restriction digest analysis and sequencing of the region of interest.

### NRPS module/domain swap technique

The previously described *aac(3)IV*-MRS-ISceI cassette was essential for all module/domain swaps. Firstly, cassette amplification was conducted with primers introducing overhangs complementary to the regions flanking the sequence which was to be exchanged. For *pac15* primers *pac15*_KO_773_f and *pac15*_KO_773_r, for *pac14* primers p*ac14*_KO_773_f and p*ac14*_KO_773_r, for *pac2* primers KO_*pac2*_FW and KO_*pac2*_RV, and for *pac12* primers *pacL*-K.O.-apra_F and *pacL*-K.O.-apra_R were used. Thus, the corresponding genes were knocked out by Red/ET-mediated homologous recombination. The resulting cosmids were linearized by I-SceI restriction and utilized as one of the two fragments for Red/ET-mediated recombination, as previously described. The other fragment was assembled by Gibson assembly. More specifically, the desired gene of the napsamycin BGC was amplified by PCR with the following primers: *npsP8*_f and *npsP8*_r for *npsP8*,* npsP4*_f and *npsP4*_r for *npsP4*,* npsN*_F and *npsN*_R for *npsN*, and *npsP3*_TF1 and *npsP3*_R for *pac12*. For each swap, two more fragments were amplified by PCR, which consist of a 300–500 bp sequence of the borders of the *aac(3)IV*-MRS-ISceI insertion site (primers: Bluescript-left_*npsP8*_f + *npsP8*-left_r and *npsP8*-right_f + Bluescript-right_*npsP8*_r for *npsP8*; Bluescript-left_*npsP4*_f + *npsP4*-left_r and *npsP4*-right_f + Bluescript-right_*npsP4*_r for *npsP4; npsN*_500L_F + *npsN*_500L_R and *npsN*_500RL_F + *npsN*_500R_R for *npsN*, and *pacL*_500L-F_SwaI + *pacL*_500_L_R-Over and *pacL*_500_R_F + *pacL*_500R-R_SwaI for *pac12*. All three purified PCR products were assembled in pBluescript SK (-), which had been linearized with EcoRV, by Gibson assembly. The assembled fragment was then excised out of the vector and purified. Once both components, the linearized cosmid and the gene to be knocked in including the necessary overhangs, were obtained, recircularization and verification were performed, as previously described.

### UPA production and extraction

For UPA production, TSB (3 mL, Sigma Aldrich, St. Louis, MO, USA) containing the appropriate antibiotics was inoculated with a spore suspension of *S. coeruleorubidus* NRRL 18370 or of a heterologous *Streptomyces* host strain harboring one of the cosmids constructed herein and potentially additional plasmids. Three independent biological replicates corresponding to three spore stocks were utilized to inoculate three precultures per strain. These precultures were incubated at 30 °C and 200 rpm for two days, before adjusting the wet cell weight of all cultures with TSB to equal concentrations. Subsequently, the production medium (3 mL) was inoculated with the adjusted precultures (50 µL) in 24-square deep-well plates and grown at 30 °C and 200 rpm for seven days. If non-integrative genetic constructs were present in the bacterial strains, appropriate antibiotics were added to the main culture. If required, sterile stock solutions of amino acids were prepared and added to the main culture prior to cultivation. For partial purification, the main cultures (700 µL) were acidified to pH 4 with HCl and extracted twice with an equal volume of *n*-butanol. The solvent was evaporated in a centrifugal vacuum concentrator and the residue dissolved in a water/methanol mixture (1:1 (*v*/*v*), 100 µL). Insoluble particles were removed by centrifugation, and the resulting crude extract was analyzed by HPLC–ESI–MS or –MS/MS analysis. Simultaneously, after seven days of incubation, 1 mL of main culture was transferred to pre-weighed tubes to determine the cell dry weight. Cells were harvested by centrifugation, washed with ddH_2_O, and centrifuged again, before drying at 80 °C for at least two days. UPA production was normalized to the resulting cell dry weights.

### mRNA isolation and sequencing

For mRNA sequencing, cultures of mutant strains were prepared, as described above, but at a larger scale. More specifically, precultures were grown in 50 mL of TSB and main cultures in 70 mL of medium P, both in baffled Erlenmeyer flasks (300 mL). After four days, the cell pellets were harvested by centrifugation (4000 rpm, 4 °C, 20 min) and shipped on dry ice to Vertis Biotechnologie AG (Freising, Germany). There, mRNA isolation and analysis, rRNA depletion, cDNA synthesis, and sequencing (Illumina NextSeq 500 system) were conducted. RNA sequencing data assessment and analysis were conducted, as previously reported [48].

### Analysis of UPA production by HPLC-ESI-MS and MS/MS

UPA production was investigated utilizing an HPLC Infinity 1200/MSD Ultra Trap System XCT 6330 (Agilent Technologies, Santa Clara, CA, USA) equipped with a Nucleosil-100 C18 column (3 μm, 100 × 2 mm). A sample volume of 5 µL was injected and separated at a flow rate of 0.4 mL/min with a linear gradient of 20 min from 0% to 100% solvent B, followed by an isocratic step consisting of 100% solvent B for 3 min (solvent A: 0.1% formic acid in LC–MS grade ddH_2_O, solvent B: 0.06% formic acid in LC–MS grade acetonitrile). The mass spectrometer was equipped with an ESI interface (capillary voltage: 3.5 kV, heated capillary temperature: 350 °C), where positive ionization was performed.

### Agar diffusion bioassays

Main cultures of different heterologous *Streptomyces* host strains and the native producer strain *S. coeruleorubidus* NRRL 18370 were examined regarding their bioactivity, which corresponds to UPA production, in agar diffusion assays. Bioassay plates were prepared by adding 100 µL of an overnight culture of *E. coli* BW25113Δ*tolC* to 100 mL LB agar, immediately before to pouring the plates. The strains to be tested were cultivated in accordance with the abovementioned methods. Part of each main culture (1 mL) was extracted, as described above. The extracts were dissolved in 100 µL DMSO and 10 µL of extract was applied to filter disks (4 mm) placed on the previously prepared agar plates. After incubating the obtained plates for 24 h at 37 °C, the zone of inhibition was measured.

### MIC determination of UPAs

Minimal inhibitory concentration (MIC) was determined by broth microdilution, according to CLSI guidelines, in polypropylene 96-well microtiter plates (Nunc brand) using Luria-Bertani broth (LB). The respective compounds were diluted to reach 128 µg/mL in the first well and were subsequently serial diluted. The inoculum was prepared from a liquid preculture of *P. aeruginosa* PA01, grown to midlog phase and diluted to reach a final concentration of 5 × 10^5^ cfu/mL in the wells. The plate was incubated for 24 h and MICs were determined as the lowest concentrations where no visible growth was observed.

### IC_50_ determination of UPAs

For the isolation of dansylated UDP-MurNAc pentapeptide, 1 mM of the purified UDP-MurNAc pentapeptide was mixed with 42 mM dansylchloride in acetone and 0.25 M NaHCO_3_ in a ratio of 1:1 (vol/vol). The reaction was stirred overnight at room temperature and acetone was evaporated. The resulting aqueous phase was injected into the HPLC system. Due to the differences in charge at alkaline pH, the dansylated form could be separated from the non-dansylated and remaining dansylchloride in a single-step HPLC purification using a DEAE column. 0.1 M NH_4_CO_3_ (pH 8.5) was increased to 0.5 M NH_4_CO_3_ (pH 8.5) over 35 min, followed by a 5-min wash and a re-equilibration to the initial conditions. The dansylated UDP-MurNAc pentapeptide was identified by fluorescence (Ex.: 325 nm; Em.: 495 nm) and UV absorbance at 262 nm. The concentration was determined from the peak area at 262 nm using a calibration curve of a standard of known concentration.

The construct encoding *P. aeruginosa* PAO1 *mraY* with an N-terminal His6-tag and thrombin cleavage site was generated by In-Fusion Cloning (Takara Bio) according to the manufacturer’s instructions. The insert *mraY* was amplified by PCR using the forward primer 5’-CGCGGCAGCCATATGCTCCTGCTGCTGGCCGAA-3’ and reverse primer 5’- GTGGTGGTGCTCGAGTCAACGCAGCTTCAAGGTGG-3’. The vector pET28a was amplified using the forward primer 5’-CTCGAGCACCACCACCACC-3’ and the reverse primer 5’-CATATGGCTGCCGCGCGG-3’. Chemically competent *Escherichia coli* BL21 C43 (DE3) cells were transformed with the resulting plasmid. Protein expression and purification were performed as described previously [49, 50] with minor modifications. Cells were grown in 2YT medium containing 50 µg/mL kanamycin at 30 °C to an OD600 of 0.6. Protein expression was induced with 1 mM IPTG, followed by incubation for 16 h at 20 °C. Cells were harvested (10,000 x g, 12 min, 4 °C) and washed in 25 mM Tris-HCl, pH 7.5. Pellets were resuspended in buffer A (25 mM Tris-HCl, pH 7.5, 2 mM 2-mercaptoethanol, 150 mM NaCl, 10% glycerol, and 1 mM MgCl_2_). 1 mg/mL lysozyme, 5 µg/mL DNase, and 10 µg/mL RNase were added, followed by incubation on ice for 45 min and sonication. Lysates were centrifuged (20,000 x g, 30 min, 4 °C) and membrane pellets were solubilized overnight (4 °C) in buffer A containing 21.5 mM *n*-dodecyl-β-D-maltoside (DDM). Following centrifugation (20,000 x g, 30 min, 4 °C), the supernatant was purified by Ni-NTA affinity chromatography. The resin was transferred to polypropylene columns (Thermo Fisher) and washed with buffer A followed by buffer B (buffer A supplemented with 3.9 mM DDM). Proteins were eluted stepwise with buffer B containing increasing concentrations of imidazole (100 mM, 200 mM, and 300 mM). Protein purity was assessed by SDS-PAGE, and concentrations were determined by Bradford assay (Bio-Rad Laboratories). Purified protein fractions were stored at -20 °C.

For the fluorescence-based assay, 0.5 µg of purified MraY-His6 was incubated with 50 mM Tris/HCl (pH 8.0), 0.1% Triton X-100, 200 mM KCl, 50 mM MgCl_2_, 50 µM C55P, 8% glycerol, and 25 µM UDP-MurNAc l-Ala-d-Glu-*m*-DAP-(N_ε_-dansyl)-d-Ala-d-Ala, in a black flat-bottom 384-well plate. The formation of dansylated lipid I was monitored in a spectrofluorometer (Ex.: 325 nm; Em.: 525 nm). IC_50_ values were determined by plotting the activity against the inhibitor concentration.

## Supplementary Information

Below is the link to the electronic supplementary material.

Supplementary Material 1 Additional Figures S1 to S17 and Tables S1 to S6 can be found in additional file 1 (.docx).

## Data Availability

The datasets generated and/or analyzed during this study are included in this article and its supplementary information files. The constructed mutant strains, cosmids, and plasmids are available at the Department of Pharmaceutical Biology of the University of Tübingen, Tübingen, Germany. The mRNA sequencing data discussed in this publication have been deposited in NCBI’s Gene Expression Omnibus [51] and are accessible through GEO Series accession number GSE333483 (https://www.ncbi.nlm.nih.gov/geo/query/acc.cgi?acc=GSE333483).
