# Supplementary material for: Engineering a production platform for the potent antipseudomonal antibiotic mureidomycin A by reprogramming the pacidamycin biosynthetic gene cluster
Source: Microb Cell Fact. 2026 Oct 30;25:227. doi: 10.1186/s12934-026-03166-9 (PMC13721732; doi:10.1186/s12934-026-03166-9)
Supplement: Supplementary file 1 [file 12934_2026_3166_MOESM1_ESM.docx]

**Additional File 1 for**

**Engineering a Production Platform for the Potent Antipseudomonal Antibiotic Mureidomycin A by Reprogramming the Pacidamycin Biosynthetic Gene Cluster**

Panagiota-Hanna Koutsandrea^#,1,2^, Richard Sonnengrün^#,1,2^, Nils Hauck^1^, Andreas Kulik^3^, Theresa Harbig^4^, Jan-Martin Daniel^6,7^, Kay Katja Nieselt^4^, Rebecca Jane Miriam Goss^5^, Tanja Schneider^6,7^, Silja Mordhorst^1,2^, Harald Gross^1,2^ and Bertolt Gust^1,2*^

^1^Department of Pharmaceutical Biology, Eberhard Karls University Tübingen, Auf der Morgenstelle 8, 72076 Tübingen, Germany

^2^German Center for Infection Research (DZIF), Partner Site Tübingen, 72076 Tübingen, Germany

^3^Department of Microbial Bioactive Compounds, Interfaculty Institute of Microbiology and Infection Medicine, Eberhard Karls University Tübingen, Auf der Morgenstelle 28, 72076 Tübingen, Germany

^4^Institute for Bioinformatics and Medical Informatics, Eberhard Karls University Tübingen, Sand 14, 72076 Tübingen, Germany

^5^School of Chemistry, Institute of Engineering, University of St Andrews, St Andrews KY16 9ST, United Kingdom

^6^Institute for Pharmaceutical Microbiology, University of Bonn, University Hospital Bonn, 53127 Bonn, Germany

^7^German Center for Infection Research (DZIF), Partner Site Bonn-Cologne, 53113 Bonn, Germany

*Corresponding author: bertolt.gust@uni-tuebingen.de

^#^These authors contributed equally.

**Table of contents:**

- **Table S1:** Structures of selected uridyl peptide antibiotics. (Pages 3–4)
- **Table S2:** Primers used in this study. (Pages 4–6)
- **Table S3:** Influence on production of pacidamycin D and S (and T) by feeding *S. albus* J1074/pacNH01 with different amino acids. (Page 6)
- **Figure S1:** Overview of the biosynthetic gene clusters of this study and the main products formed by each one. (Page 7)
- **Figure S2:** Production of pacidamycins in wild-type and heterologous strains. (Page 8)
- **Figure S3:** LC–MS/MS analysis of Met-pacidamycin S (*m*/*z* 733.3 [M+H]^+^) production in *S. albus* J1074/ pacNH01_*npsP8P4*. (Page 8)
- **Figure S4:** LC–MS/MS analysis of Met-pacidamycin D (*m*/*z* 772.3 [M+H]^+^) production in *S. albus* J1074/ pacNH01_*npsP8P4*. (Page 9)
- **Figure S5:** Comparison of UPA production before and after *pac15*/*npsP8* and *pac14*/*npsP4* swaps by LC–MS analysis. (Page 9)
- **Table S4:** Organization and sequences of synthetic promoters used in the present study. (Page 10)
- **Figure S6:** RNA sequencing data of the native pacidamycin gene cluster of strain *S. albus* J1074/pacNH01 after 4 days of cultivation. (Page 11)
- **Figure S7:** RNA sequencing data of the modified pacidamycin gene cluster of strain *S. albus* J1074/pacRS01 after 4 days of cultivation. (Page 12)
- **Figure S8:** RNA sequencing data of the modified pacidamycin gene cluster of strain *S. albus* J1074/pRS02/pacRS01 after 4 days of cultivation. (Page 13)
- **Figure S9:** RNA sequencing data of the modified pacidamycin gene cluster of strain *S. albus* J1074/pRS02/pacHK03 after 4 days of cultivation. (Page 14)
- **Figure S10:** Investigation of culture extracts of A) *S. albus* J1074/pacRS02 and B) *S. albus* J1074/pacRS02/pRS02. (Page 15)
- **Figure S11:** LC–MS/MS analysis of Met-pacidamycin 4 (*m*/*z* 864.3 [M+H]^+^) production in *S. albus* J1074/ pacRS02/pRS04. (Page 16)
- **Figure S12:** LC–MS/MS analysis of Met-pacidamycin 5 (*m/z* 825.3 [M+H]^+^) production in *S. albus* J1074/ pacRS02/pRS04. (Page 16)
- **Figure S13:** LC–MS/MS analysis of mureidomycin A (*m*/*z* 841.3 [M+H]^+^) production in *S. albus* J1074/ pacRS02/pRS04. (Page 17)
- **Figure S14:** UPA production in *S. albus* J1074/pacRS02/pRS04. (Page 17)
- **Figure S15:** Investigation of culture extracts of *S. albus* J1074/pacRS03/pRS04. (Page 18)
- **Figure S16:** Investigation of culture extracts of 1) *S. albus* J1074/pacHK11/pRS04 and 2) *S. albus* J1074/ pacRS03/pRS04 by LC–MS. (Page 18)
- **Figure S17:** Investigation of mureidomycin A production in *S*. *albus* J1074/pacHK11/pRS04 and the derived *S*. *albus* B2P1/pacHK11/pRS04, which harbors an additional *attB*(ϕC31) site. (Page 19)
- **Figure S18:** IC50 of UPA’s against P. aeruginosa MraY WT (Page 19)
- **Table S5:** Plasmids, cosmids, and bacterial strains. (Pages 20–21)
- **Table S6:** gBlocks™ of this study. (Pages 21–22)
- **References.** (Page 23)

**Table S1:** Structures of selected uridyl peptide antibiotics. The three-letter code is used for amino acid abbreviations. Bicyclic moieties are depicted below. MW = molecular weight; Met^SO^: methionine sulfoxide; *m*‑Tyr = *meta*-tyrosine.

| **Name** | **R1** | **AA2** | **AA4** | **AA5** | **Other** | **MW (Da)** |
| --- | --- | --- | --- | --- | --- | --- |
| Pacidamycin 1 | Ala | *m*-Tyr | Ala | Trp | Uracil | 874.4 |
| Pacidamycin 2 | Ala | *m*-Tyr | Ala | Phe | Uracil | 835.4 |
| Pacidamycin 3 | Ala | *m*-Tyr | Ala | *m*-Tyr | Uracil | 851.4 |
| Pacidamycin 4 | H | *m*-Tyr | Ala | Trp | Uracil | 803.3 |
| Pacidamycin 4N | H | Bicyclic a^a^ | Ala | Trp | Uracil | 815.3 |
| Pacidamycin 5 | H | *m*-Tyr | Ala | Phe | Uracil | 764.3 |
| Pacidamycin 5T | H | *m*-Tyr | Ala | *m*-Tyr | Uracil | 780.3 |
| Pacidamycin 6 | Gly | *m*-Tyr | Ala | Trp | Uracil | 860.4 |
| Pacidamycin 7 | Gly | *m*-Tyr | Ala | Phe | Uracil | 821.3 |
| Pacidamycin D | H | Ala | Ala | Trp | Uracil | 711.3 |
| Pacidamycin S | H | Ala | Ala | Phe | Uracil | 672.3 |
| Pacidamycin T | H | Ala | Ala | *m*-Tyr | Uracil | 688.3 |
| Met-pacidamycin D | H | Ala | Met | Trp | Uracil | 771.3 |
| Met-pacidamycin S | H | Ala | Met | Phe | Uracil | 732.3 |
| Met-pacidamycin 4 | H | *m*-Tyr | Met | Trp | Uracil | 863.4 |
| Met-pacidamycin 5 | H | *m*-Tyr | Met | Phe | Uracil | 824.3 |
| Mureidomycin A | H | *m*-Tyr | Met | *m*-Tyr | Uracil | 840.3 |
| Mureidomycin B | H | *m*-Tyr | Met | *m*-Tyr | Dihydrouracil | 842.3 |
| Mureidomycin C | Gly | *m*-Tyr | Met | *m*-Tyr | Uracil | 897.3 |
| Mureidomycin D | Gly | *m*-Tyr | Met | *m*-Tyr | Dihydrouracil | 899.3 |
| Mureidomycin E | H | Bicyclic a^a^ | Met | *m*-Tyr | Uracil | 852.3 |
| Napsamycin A | H | Bicyclic b^a^ | Met | *m*-Tyr | Uracil | 852.3 |
| Napsamycin B | H | Bicyclic c^a^ | Met | *m*-Tyr | Uracil | 866.3 |
| Napsamycin C | H | Bicyclic b^a^ | Met | *m*-Tyr | Dihydrouracil | 854.3 |
| Napsamycin D | H | Bicyclic c^a^ | Met | *m*-Tyr | Dihydrouracil | 868.3 |
| Sansanmycin A | H | *m*-Tyr | Met | Trp | Uracil | 863.4 |
| Sansanmycin B | H | *m*-Tyr | Leu | Trp | Uracil | 845.4 |
| Sansanmycin C | H | *m*-Tyr | Met^SO^ | Trp | Uracil | 879.4 |
| Sansanmycin D | H | *m*-Tyr | Met | *m*-Tyr | Uracil | 840.3 |
| Sansanmycin E | H | *m*-Tyr | Met^SO^ | *m*-Tyr | Uracil | 856.3 |
| Sansanmycin F | H | Bicyclic d^a^ | Met | Trp | Uracil | 903.4 |
| Sansanmycin G | H | Bicyclic d^a^ | Leu | Trp | Uracil | 885.4 |

| ^a^Bicyclic moieties: | | | |
| --- | --- | --- | --- |
|  |  |  |  |
| Bicyclic a | Bicyclic b | Bicyclic c | Bicyclic d |

**Table S2:** Primers used in this study.

| **Primer** | **Sequence 5’ 🡪 3’** | **Note** |
| --- | --- | --- |
| pacST_f | CGAGATCGAGCAGGTCGTACG | Used for the verification of the integration of pacNH01 into the heterologous host strains |
| pacST_r | CGGTCGATCATGGAGGTCAGC |  |
| pUWL_F | ACGCCTGGTCGATGTCGGAC | Used for the verification of pUWL-*aac(3)IV*_*oriT* _*pac1*, pUWL-*aac(3)IV*_*oriT* _*npsM*, and pUWL-*aac(3)IV*_*oriT* _*ssaA* |
| pUWL_R | GAGCGAGGAAGCGGAAGAGC |  |
| *pac15*_KO_773_f | GAAGCGTCGAGCCATCCTGAAACTCTGAGAGGCAAACCTTGTAGGCTGGAGCTGCTTC | Used for the amplification of *aac(3)IV*-MRS-ISceI in the swap of *pac15* |
| *pac15*_KO_773_r | GACGTACCTGTATACGCGCCATCCTGAACGACAGATGAAATTCCGGGGATCCGTCGACC |  |
| *npsP8*_f | GGAAGCGTCGAGCCATCCTGAAACTCTGAGAGGCAAACCTATGACCGCCGCAGAGCAGCG | Used for the amplification of *npsP8* in the swap of *pac15* |
| *npsP8*_r | CGACGTACCTGTATACGCGCCATCCTGAACGACAGATGAATCAGGGGCGGGGAGCTCGACG |  |
| Bluescript-left_ *npsP8*_f | CTCGAGGTCGACGGTATCGATAAGCTTGATACCGGGTCTCCTTTTTGATACGACTAGCGT | Used for the amplification of one border of *npsP8* in the swap of *pac15* |
| *npsP8*-left_r | GAGCAGAGACCGCTGCTCTGCGGCGGTCATAGGTTTGCCTCTCAGAGTTTCAGGATGGC |  |
| *npsP8*-right_f | GCAACTCCGCGTCGAGCTCCCCGCCCCTGATTCATCTGTCGTTCAGGATGGCGCGTATAC | Used for the amplification of the other border of *npsP8* in the swap of *pac15* |
| Bluescript-right_ *npsP8*_r | ACTAGTGGATCCCCCGGGCTGCAGGAATTCGATAGTGCCAGCCTTTCCGGCCGAACGTAG |  |
| p*ac14*_KO_773_f | TGTATTTGTACTTGGTCATTTTCTTTCAGCTCGTCGTCATGTAGGCTGGAGCTGCTTC | Used for the amplification of *aac(3)IV*-MRS-ISceI in the swap of *pac14* |
| p*ac14*_KO_773_r | TACTCGACGCTAGTCGTATCAAAAAGGAGACCCGGTGTGATTCCGGGGATCCGTCGACC |  |
| *npsP4*_f | GTGTTTCCGCTCAGCGCCGCA | Used for the amplification of *npsP4* in the swap of *pac14* |
| *npsP4*_r | TCAGCGCCCCGCCATCGC |  |
| Bluescript-left_ *npsP4*_f | CTCGAGGTCGACGGTATCGATAAGCTTGATGCCATCCTCCATCCAGCTGGTGGTGATGCC | Used for the amplification of one border of *npsP4* in the swap of *pac14* |
| *npsP4*-left_r | GCGGCGACGGCCGCGATGGCGGGGCGCTGACGACGAGCTGAAAGAAAATGACCAAGTACA |  |
| *npsP4*-right_f | GATTTGCTGTGCGGCGCTGAGCGGAAACACACCGGGTCTCCTTTTTGATACGACTAGCGT | Used for the amplification of the other border of *npsP4* in the swap of *pac14* |
| Bluescript-right_ *npsP4*_r | ACTAGTGGATCCCCCGGGCTGCAGGAATTCGATAGGTTTGCCTCTCAGAGTTTCAGGATG |  |
| KO_*pac2*_FW | CTCGCTGGTTGGACACTAGATCATGAGGGCTGATACATGATTCCGGGGATCCGTCGACC | Used for the amplification of *aac(3)IV*-MRS-ISceI in the swap of *pac2* |
| KO_*pac2*_RV | CCCGGTGGCTGCCGGCGGGTACCAGCCGCCTCCCTCTTATGTAGGCTGGAGCTGCTTC |  |
| *npsN*_F | GGTTGGACACTAGATCATGAGGGCTGATACATGGCCATGGGCTTCACCGC | Used for the amplification of *npsN* in the swap of *pac2* |
| *npsN*_R | TCATGAGCCGGCGCGCGCGAGG |  |
| *npsN*_500L_F | AGTGGATCCCCCGGGCTGCAGGAATTCGATATCCTTCATGGTCTACAACGTCG | Used for the amplification of one border of *npsN* in the swap of *pac2* |
| *npsN*_500L_R | GTATCAGCCCTCATGATCTAGTGTCC |  |
| *npsN*_500R_F | ACACGCGCCCTCGCGCGCGCCGGCTCATGAGAGGGAGGCGGCTGGTACCC | Used for the amplification of the other border of *npsN* in the swap of *pac2* |
| *npsN*_500R_R | CCCCTCGAGGTCGACGGTATCGATAAGCTTGATATCGAGATGTCGCGCGTCCTGATCC |  |
| BidPrCas1_FW_vol2 | TACTTCAGTCACGGCGCCAACGTAGCAGGATGGGTTCAATGAGGACCACCTCTCATAAC | Used for the amplification of PC1 for the construction of pacHK03 |
| BidPrCas1_RV | GGTCAGTTGTGCGCTCAACAAGGGAAGCCTGTCGGTCATAGCGGACCTCTATGGCTTG |  |
| neu_*pac3*_test_FW | CAATCATGATGGAGCGCTTG | Used for the verification of pacHK03 and the bacterial strains harboring pacHK03 |
| *pac4*_test_RV | ATGCACGGAACATCAAGGTG |  |
| pMVM3100_*pac21h*_F | AAAAACTAGTAACGCTCACTGGGTACCAGC | Used for the construction of pRS02 |
| pMVM3100_*pac21h*_R | CCAAGCTTGGCTCAGTGAGG |  |
| pUWL-P*ermE*+*npsM*_F | AAAAAACCTAGGACTTCGTGCAGGCGGTAC | Used for the construction of pRS04 |
| pUWL-P*ermE*+*npsM*_R | AAAAAACCTAGGCTAGTTCATGAGATCGCGCC |  |
| *PacU*_KO_F | TGCCTGGCTTCCGTCGCCTTACTGATGCCGACGCCTTGCATTCCGGGGATCCGTCGACC | Used for the knockout of *pac21* |
| *PacU*_KO_R | AATCATTTCGCAGCCGAAGGCCGGGGGTTCGCAAGGTTTTGTAGGCTGGAGCTGCTTC |  |
| *pacL*-K.O.-apra_F | ATGGGAGCGGGCATCACCACCAGCTGGATGGAGGATGGCATTCCGGGGATCCGTCGACC | Used for the amplification of *aac(3)IV*-MRS-ISceI in the swap of *pac12* |
| *pacL*-K.O.-apra_R | GGGGCGGCGCCTTTTCCATCGCAAACGCGTGGCACGAGGTGTAGGCTGGAGCTGCTTC |  |
| *npsP3*_TF1 | ATGGAGGATGGCAAGTCGATG | Used for the amplification of *npsP3* in the swap of *pac12* |
| *npsP3*_R | TCAGAGCAGCCGCGCGAGCTC |  |
| *pacL*_500L-F_SwaI | AAAAATTTAAATCTCGTTGCCGCGAGAACTGG | Used for the amplification of one border of *npsP3* in the swap of *pac12* |
| *pacL*_500_L_R-Over | CATCGACTTGCCATCCTCCATGCCATCCTCCATCCAGCTGG |  |
| *pacL*_500_R_F | ACCGTCGCCGAGCTCGCGCGGCTGCTCTGACCTCGTGCCACGCGTTTGCG | Used for the amplification of the other border of *npsP3* in the swap of *pac12* |
| *pacL*_500R-R_SwaI | TTTTATTTAAATGCGGACAACGCCTTCCTCTC |  |

| ISceI_pacRS03_PS1_FW | GGTCAGTTGTGCGCTCAACAAGGGAAGCCTGTCGGTCATATTCCGGGGATCCGTCGACC | Used for the amplification of the apramycin cassette in the construction of pacHK11 |
| --- | --- | --- |
| ISceI_pacRS03_PS1_RV | TACTTCAGTCACGGCGCCAACGTAGCAGGATGGGTTCAATGTAGGCTGGAGCTGCTTC |  |
| PC1_on_RS03_Amp_FW | ATGAGCAGCATCGACTGTTC | Used for the amplification of promoter cassette 1 out of pacHK03 in the construction of pacHK11 |
| PC1_on_RS03_Amp_RV | CAGGTAGCTCTGGTAAGCG |  |

**Table S3:** Influence on production of pacidamycin D and S (and T) by feeding *S. albus* J1074/pacNH01 with different amino acids. Concentrations were calculated *via* peak area integration of HPLC chromatograms using pacidamycin 4 as a standard; n.d. = not detected.

| **Precursor** | **Pacidamycin D** | **Pacidamycin S** | **Pacidamycin T** |
| --- | --- | --- | --- |
| None | 52.43 ± 1.50 mg/L | 29.24 ± 1.49 mg/L | n.d. |
| 0.5 mM l-alanine | 68.20 ± 2.46 mg/L | 37.22 ± 2.21 mg/L | n.d. |
| 1.0 mM l-alanine | 77.65 ± 1.03 mg/L | 41.20 ± 0.86 mg/L | n.d. |
| 2.0 mM l-alanine | 79.66 ± 2.23 mg/L | 43.61 ± 1.92 mg/L | n.d. |
| 0.5 mM *m*-tyrosine | 47.82 ± 1.67 mg/L | 19.30 ± 1.76 mg/L | 10.31 ± 0.71 mg/L |
| 1.0 mM *m*-tyrosine | 37.26 ± 2.71 mg/L | 16.81 ± 0.76 mg/L | 15.32 ± 1.34 mg/L |
| 2.5 mM *m*-tyrosine | 29.29 ± 2.54 mg/L | 13.42 ± 1.11 mg/L | 18.65 ± 1.46 mg/L |
| 5.0 mM *m*-tyrosine | 22.14 ± 0.98 mg/L | 11.84 ± 0.50 mg/L | 27.12 ± 0.82 mg/L |
| 10.0 mM *m*-tyrosine | 10.09 ± 0.94 mg/L | 8.14 ± 0.83 mg/L | 31.66 ± 1.03 mg/L |

**Figure S1:** Overview of the biosynthetic gene clusters of this study and the main products formed by each one (right side). The gene clusters are depicted top–down in order of construction. The modifications that are introduced are marked, while the unmodified genes are transparent. PCD = pacidamycin, M-PCD = Met-pacidamycin, SAN = sansanmycin, MRD = mureidomycin.

**Figure S2:** Production of pacidamycins in wild-type and heterologous strains. Extracted ion chromatograms (EICs) of pacidamycin D (*m*/*z* 712 [M+H]^+^, left side) and pacidamycin S (*m*/*z* 673 [M+H]^+^, right side) in the wild-type producer *S. coeruleorubidus* NRRL 18370 (green, 4) and in the heterologous hosts *S. coelicolor* M1146 (blue, 3), *S. coelicolor* M1154 (red, 2), and *S. albus* J1074 (black, 1) harboring the pacidamycin biosynthetic gene cluster of pacNH01.

**Figure S3:** LC–MS/MS analysis of Met-pacidamycin S (*m*/*z* 733.3 [M+H]^+^) production in *S. albus* J1074/ pacNH01_*npsP8P4*. Top: Base peak chromatogram (+MS 650–950 *m*/*z*) and chemical structure of Met-pacidamycin S with possible fragment masses. Bottom: MS/MS fragmentation pattern of Met-pacidamycin S (*m*/*z* 733.3 [M+H]^+^).

**Figure S4:** LC–MS/MS analysis of Met-pacidamycin D (*m*/*z* 772.3 [M+H]^+^) production in *S. albus* J1074/ pacNH01_*npsP8P4*. Top: Base peak chromatogram (+MS 650–950 *m*/*z*) and chemical structure of Met-pacidamycin D with possible fragment masses. Bottom: MS/MS fragmentation pattern of Met-pacidamycin D (*m*/*z* 772.3 [M+H]^+^).

**Figure S5:** Comparison of UPA production before and after *pac15*/*npsP8* and *pac14*/*npsP4* swaps by LC–MS analysis. Extracted ion chromatograms (EICs) of LC–MS runs performed in positive mode on crude extracts of: top) *S. albus* J1074/pacNH01 extracted for pacidamycin D (*m*/*z* 712 [M+H]^+^) and pacidamycin S (*m*/*z* 673 [M+H]^+^), middle) *S. albus* J1074/pacNH01_*npsP8P4* extracted for pacidamycin D (*m*/*z* 712 [M+H]^+^) and pacidamycin S (*m*/*z* 673 [M+H]^+^), and bottom) *S. albus* J1074/pacNH01_*npsP8P4* extracted for Met‑pacidamycin D (*m*/*z* 772 [M+H]^+^) and Met-pacidamycin S (*m*/*z* 733 [M+H]^+^).

**Table S4:** Organization and sequences of synthetic promoters used in the present study. RBS = ribosome binding site.

| **Name** | **Promoter sequence (45 bp)** | **Insulation (15 bp)** | **RBS (13–16 bp)** |
| --- | --- | --- | --- |
| P15 | AATTGCCCACTTGACGTTGAGAGTGAAGCAATATAGGTTAACCT | GCCACGTGTTCGTTA | TGAGAGGTGGTCCTCA |
| P16 | GGTGATCAGTTTGACGCCCCTGACATCGGGTGGTAGCGTCGCTG | GTGGTCCGTCAAGCC | ATAGAGGTCCGCT |

**Figure S6:** RNA sequencing data of the native pacidamycin gene cluster of strain *S. albus* J1074/pacNH01 after 4 days of cultivation. The annotated gene cluster is displayed with arrows on the x-axis in the middle of the graph. While the gene cluster is separated into sense and antisense strand, the y-axis depicts the coverage plots for each strand (>0 is sense, <0 is antisense).

**Figure S7:** RNA sequencing data of the modified pacidamycin gene cluster of strain *S. albus* J1074/pacRS01 after 4 days of cultivation. The annotated gene cluster is displayed with arrows on the x-axis in the middle of the graph. While the gene cluster is separated into sense and antisense strand, the y-axis depicts the coverage plots for each strand (>0 is sense, <0 is antisense).

**Figure S8:** RNA sequencing data of the modified pacidamycin gene cluster of strain *S. albus* J1074/pRS02/pacRS01 after 4 days of cultivation. The annotated gene cluster is displayed with arrows on the x-axis in the middle of the graph. While the gene cluster is separated into sense and antisense strand, the y-axis depicts the coverage plots for each strand (>0 is sense, <0 is antisense).

**Figure S9:** RNA sequencing data of the modified pacidamycin gene cluster of strain *S. albus* J1074/pRS02/pacHK03 after 4 days of cultivation. The annotated gene cluster is displayed with arrows on the x-axis in the middle of the graph. While the gene cluster is separated into sense and antisense strand, the y-axis depicts the coverage plots for each strand (>0 is sense, <0 is antisense).

**Figure S10:** Investigation of culture extracts of A) *S. albus* J1074/pacRS02 and B) *S. albus* J1074/pacRS02/pRS02. Top to bottom: Extracted ion chromatograms (EICs) of Met-pacidamycin D (*m*/*z* 772.3 [M+H]^+^), Met-pacidamycin S (*m*/*z* 733.2 [M+H]^+^), Met-pacidamycin 4 (1, *m*/*z* 864.3 [M+H]^+^), Met-pacidamycin 5 (2, *m*/*z* 825.3 [M+H]^+^), and mureidomycin A (3, *m/z* 841.3 [M+H]^+^). UPA production is completely abolished in *S.* *albus* J1074/pacRS02 (A).

**Figure S11:** LC–MS/MS analysis of Met-pacidamycin 4 (*m*/*z* 864.3 [M+H]^+^) production in *S. albus* J1074/ pacRS02/pRS04. Top: Chemical structure of Met-pacidamycin 4 with possible fragments. Bottom: MS/MS fragmentation pattern of Met-pacidamycin 4.

**Figure S12:** LC–MS/MS analysis of Met-pacidamycin 5 (*m/z* 825.3 [M+H]^+^) production in *S. albus* J1074/ pacRS02/pRS04. Top: Chemical structure of Met-pacidamycin 5 with possible fragments. Bottom: MS/MS fragmentation pattern of Met-pacidamycin 5.

**Figure S13:** LC–MS/MS analysis of mureidomycin A (*m*/*z* 841.3 [M+H]^+^) production in *S. albus* J1074/ pacRS02/pRS04. Top: Chemical structure of mureidomycin A with possible fragments. Bottom: MS/MS fragmentation pattern of mureidomycin A.

**Figure S14:** UPA production in *S. albus* J1074/pacRS02/pRS04. Extracted ion chromatograms (EICs) of LC–MS analysis performed in the positive mode of *S. albus* J1074/pacRS02/pRS04 culture extracts. Top: extracted for Met-pacidamycin S (*m/z* 733.2 [M+H]^+^) and Met-pacidamycin D (*m/z* 772.3 [M+H]^+^); bottom: extracted for Met‑pacidamycin 4 (*m/z* 864.3 [M+H]^+^), Met-pacidamycin 5 (*m/z* 825.3 [M+H]^+^), and mureidomycin A (*m/z* 841.3 [M+H]^+^).

**Figure S15:** Investigation of culture extracts of *S. albus* J1074/pacRS03/pRS04. UV chromatogram (250–270 nm) and extracted ion chromatograms of (EICs, top to bottom): mureidomycin A (*m*/*z* 841.3 [M+H]^+^), Met-pacidamycin 5 (*m*/*z* 825.3 [M+H]^+^), and Met-pacidamycin 4 (*m*/*z* 864.3 [M+H]^+^).

**Figure S16:** Investigation of culture extracts of 1) *S. albus* J1074/pacHK11/pRS04 and 2) *S. albus* J1074/ pacRS03/pRS04 by LC–MS. Extracted ion chromatograms of mureidomycin A (*m/z* 841.3 [M+H]^+^).

**Figure S17:** Investigation of mureidomycin A production in *S*. *albus* J1074/pacHK11/pRS04 and the derived *S*. *albus* B2P1/pacHK11/pRS04, which harbors an additional *attB*(ϕC31) site. Extracted ion chromatograms of mureidomycin A (*m/z* 841.3 [M+H]^+^) are shown for two biological replicates of *S*. *albus* J1074/pacHK11/pRS04 (black traces) and *S*. *albus* B2P1/pacHK11/pRS04 (red traces).

**Figure S18:** IC_50_ of UPA’s against P. aeruginosa MraY WT. The assay was conducted in a 384-well format with the dansylated substrate UDP-MurNAc pentapeptide (mDAP) with a 10-step serial dilution of the respective compound in presence of 0.5 µg of protein. The IC_50_ was calculated by plots of the percent inhibition against the inhibitor concentration. All assays were performed in triplicates and 50 µg/ml tunicamycin served as control.

**Table S5:** Plasmids, cosmids, and bacterial strains.

| **Plasmids, cosmids, strains** | **Description** | **Reference** |
| --- | --- | --- |
| pIJ790 | Recombineering plasmid: λ-RED (*gam*, *bet*, *exo*), *araC*, *rep101^ts^*; Cml^R^ | Gust *et al.* [1] |
| pIJ773 | pBluescript II SK(+)-derivative, *P1*-FRT-*oriT*-*aac(3)IV*-FRT-*P2;* Apra^R^ | Gust *et al.* [1] |
| pMVM3100 | Integrative plasmid, derivative of pRT801, *attP*/*int* BT1, synthetic cassette [TermFd + promoter P57+ N12 UTR + NdeI-SpeI cloning sites + TermFd] cloned into SpeI-EcoRV sites; Apra^R^ | Martha V. Mendes, University of Porto; unpublished |
| pSG123 | pIJ10257-derivative, containing *pac21h* and *phhA* | Grüschow *et al*. [2] |
| pUWL-*aac(3)IV_oriT* | pUWL-*oriT*-derivative, constitutive P*ermE** promoter, pIJ101-origin, ColE1-origin, *oriT*; Apra^R^ Carb^R^ | Erb *et al.* [3] |
| pUWL_ *aac(3)IV_oriT_pac1* | Replicative plasmid, derivative of pUWL-*aac(3)IV_oriT*, *pac1* cloned into SpeI-EcoRV sites; Apra^R^ | This study |
| pUWL_ *aac(3)IV_oriT* _*ssaA* | Replicative plasmid, derivative of pUWL-*aac(3)IV_oriT*, *ssaA* cloned into SpeI-EcoRV sites; Apra^R^ | This study |
| pUWL_ *aac(3)IV_oriT* _*npsM* | Replicative plasmid, derivative of pUWL-*aac(3)IV_oriT*, *npsM* cloned into SpeI-EcoRV sites; Apra^R^ | This study |
| pRS02 | pMVM3100 derivative, P*ermE**_*pac21h*_*phhA* cloned into SpeI/HindIII restriction sites; Apra^R^ | This study |
| pRS04 | pRS02 derivative, P*ermE**_*npsM* cloned into EcoRV restriction site; Apra^R^ | This study |
| pUB307 | Conjugative plasmid, helper element to mobilize vectors in triparental conjugations; Kan^R^ | Flett *et al*. [4] |
| pUZ8002 | Conjugative plasmid, helper element to mobilize vectors in biparental conjugations; Kan^R^ | Paget *et al*. [5] |
| SuperCos1 | Cosmid vector; Carb^R^ Kan^R^ | Stratagene Inc., La Jolla, CA, USA |
| pacNH01 | Integrative derivative of SuperCos1-based cosmid containing the pacidamycin biosynthetic gene cluster; *bla* gene replaced by cassette from pIJ787 (*oriT, tet, attP* and *int* of phage φC31); Kan^R^ Tet^R^ | This study,  Rackham *et al.* [6],  pIJ787: Eustáquio *et al.* [7] |
| pacNH01_*npsP8P4* | pacNH01 derivative, *pac14* and *pac15* replaced by *npsP4* and *npsP8* of the napsamycin BGC, respectively; Kan^R^ Tet^R^ | This study |
| pacRS01 | pacNH01_*npsP8P4* derivative, *pac2* replaced by *npsN* of the napsamycin BGC; Kan^R^ Tet^R^ | This study |
| pacHK03 | pacRS01 derivative, intergenic region between *pac3* and *pac4* replaced by bidirectional promoter cassette; based on annotation of the pacidamycin BGC by Rackham *et al*. 2010 [6]; P15–P16 (PC1); Kan^R^ Tet^R^ | This study |
| pacRS02 | pacRS01 derivative, *pac21* knocked out; Kan^R^ Tet^R^ | This study |
| pacRS03 | pacRS02 derivative, *pac12* replaced by *npsP3* of the napsamycin BGC; Kan^R^ Tet^R^ | This study |
| pacHK11 | pacRS03 derivative, intergenic region between *pac3* and *pac4* replaced by bidirectional promoter cassette; based on annotation of the pacidamycin BGC by Rackham *et al*. 2010 [6]; P15–P16 (PC1); Kan^R^ Tet^R^ | This study |
| *E. coli* DH5α | F^-^ Φ80*lac*ZΔM15 Δ(*lac*ZYA-*arg*F)U169 *rec*A1 *end*A1 *hsd*R17(r_k_-, m_k_+) *pho*A *sup*E44 λ^-^*thi*-1 *gyr*A96 *rel*A1 | Thermo Fisher  Scientific |
| *E. coli* XL1-Blue | F^-^ *rec*A1 *end*A1 *gyr*A96 *(nal^r^) thi*-1 *hsd*R17(r_k_^-^m_k_^+^) *sup*E44 *rel*A1 *lac* [F´ *pro*AB*^+^* *lacI*^q^ZΔM15 *Tn*10 (Tet^R^)] | Stratagene Inc.,  La Jolla, CA, USA |
| *E. coli* ET12567 | *dam-13::Tn9 dcm-6 hsdM hsdR zjj202::Tn10 recF143 galK2* *galT22 ara-14 lacY1 xyl-5 leuB6 thi*-1 *tonA31 rpsL136 hisG4 tsx-78 mtl-1 glnV44*; Cml^R^ | MacNeil *et al.* [8] |
| *E. coli* BW25113 | F^-^ LAM^-^ *lacI*^+^ *rrnB*_T14_ Δ*lacZ*_WJ16_ *hsdR514* Δ*araBAD*_AH33_ Δ*rhaBAD*_LD78_ rph-1 | Datsenko and  Wanner [9,10] |
| *E. coli* BT340 | *E. coli* DH5α harboring pCP20 (*FLP*^+^ λ cI857^+^ λ p_R_ Rep^ts^); Amp^R^ Cml^R^ | Cherepanov & Wakernagel [11] |
| *S. coeruleorubidus* NRRL 18370 | Pacidamycin wild-type producer | Zhang *et al*. [12] |
| *S. coelicolor* M512 | Derivative of wild-type *S. coelicolor* A3(2); ∆*redD* Δ*act*II-ORF4 SCP1^-^ SCP2^-^ | Floriano and  Bibb [13] |
| *S. coelicolor* M1146 | Derivative of wild-type *S. coelicolor* A3(2); ∆*act* ∆*red* ∆*cpk* ∆*cda* SCP1^-^ SCP2^-^ | Gomez-Escribano and Bibb [14] |
| *S. coelicolor* M1154 | Derivative of *S. coelicolor* M1146; *rpoB* (C1298T) *rpsL* (A262G) | Gomez-Escribano and Bibb [14] |
| *S. lividans* TK24 | Derivative of *S. lividans* 66; *str-6* SLP2^-^ SLP3^-^ | Hopwood *et al*. [15] |
| *S. venezuelae* ATCC 10712 | Wild-type strain | Ehrlich *et al*. [16] |
| *S. albus* J1074 | Derivative of *S. albus* G, defective in *Sal*I restriction–modification system | Chater and Wilde [17] |
| *S*. *albus* B2P1 | Derivative of *S. albus* J1074, in which 15 specialized metabolite gene clusters have been deleted and an additional *attB*(ϕC31) site has been integrated into the genome | Myronovskyi *et al*. [18] |

**Table S6:** gBlocks™ of this study.

| **Name** | **Sequence** |
| --- | --- |
| *ssaA*_polyA_ecoRV_speI | AAAAAAGATATCATGAATACCATGACCGAATCCGATGTAGAAGTGATCCTGACCTGGCGGAACAAGACCCGCCACCACGGCAGCTCCGACGCCAAGGAACGGATCAACCTGAAGGTCGGCGAACGCCTGAACTTCGGCATATCGAGCTCCACCGGCGAATTCCAGCCCGGCGCCCTGCCGAGCATCGGAACGTTCGAAGTGTCCGACCATCACCTGCTCTTCTCGAACTTCACGACGAGCACCACGTTCGTCATCGAGAACCTGGAGGGCGGCACCGAACTGGTCAAGGCCCGCCCGCGTCAGCTGGGCATGGTGATCCCGTTCGAGATGTCGCGCATCCTGATCCCGTCCAGCGAGACCATCAGCGAACTGACGGTCTTCACTCCCCCTCCGCGCCTGCTCGGCTCCGACCAGGCCGCCACCCTCACCGAGTCCGCGATGTCCAAGCTGGACACGGCGAGCAAGTACTTCGCCGTCCTCGTGGCCCTGTGCGAGCCGCGACTGCGCGGTTCCTCCATGGCGGCCGTCCCCTCCGTGCAGGAGGTGGTCGAACGGCTGCGCGGGATCAAGCAGTTCAAGGGCGCGAACCGCAGCAGCATCAACTACCACATCGACTACCTGGCGGATCAGAAGCTTCCGGTCGGCCAGTGGGCCAAGTACGTGGACGAGGGCCGCATGCACTCCAAGCGCGAGGCCCTGGTCGCGTTCTCGCTCCGGTTCGACCTGGTCCGGGAGGAACACCTGGCGCTGCTTCCCGATCTCAAACGTGCGAGGGCACAATGTGCCTGAACTAGTTTTTTT |
| *npsM*_polyA_ecoRV_speI | AAAAAAGATATCATGGACAACCTTTTCGAGCGTGATGCAGAAGTGATTCTGACCTGGCGCGTCCAGGCCCGCCACCTGGGTGGATCCACTACCCAGGAAAGGATCAACCTGAAGGTCGGCGATCGGCTGGACTTCGGCATACCCAACGCCGGCGGAGAATTCCAGCCCCGTGCGACGCCCAGCATCGGCACTTTTGAGGTGTCCGACCAGCACCTCTTCTTCTCGAACTTCACCACCAGCACCACGTTCGTCATCGAGAATCTGGAGGGCGGGACCGAACTCGTCAAGGCGCGCCCCCGCCAGCTCGGCATGGTGATCCCGTTCGAGATGTCGCGCATCCTCATCCCCTCCAGCGCAAGCATCGGCGAACTCACCGTCTTCACATCCCCGCCCCGCCTGCTCGGGCCCGAGATGGCCGCCACGCCCACCGAGTCCGCGATGTCCAAACTGGACGCGACGACCAAGTACTTCGCCGTTCTCGTCGCCTTGTGCGAGCCGCGTCTGCGCGGTTCCTCGATGGCGGCTGTGCCCTCCGTCCAGGAGGTGGTGGAAAGACTGCGGGGGGTCGAGCAGTTCAGGGACGCCAACCGCAGCAGCGTCAACTACCACATCGACTATCTGGCTGACCAGAAGCTTCCGGTCAGCCAGTGGGCCAAGTACCTGAACGAAGGGCGCATGCATTCGAAGCGCGAGGCCCTGGTCGCCTTCTCGCTCCGCTTCGACCTGGTCCGCGAGGAACACCTCGCCCTCCTCCCCGACGTCAAACTCCTGCGCGCGCAACGATGTCTTGAGGCACGGCAAGCCACGAAGGTGGCAACGGGCGCGATCTCATGAACTAGTTTTTTT |
| *pac1*_polyA_ecoRV_speI | AAAAAAGATATCATGCACACCAAGATCGAAGCCGATGTAGAAGTGATCGTGACCTGGCGGAACAAGACCCGCCATCCTGGTGGACCCGTCGCCAAGGAACGGATCAACCTGAAGGTCGGCGATCGACTGAACTTCGGCATATCCAGCTACACCGGAGAATTCCAGCCCGGCGCTACGCCGAGCATCGGCGCGTTCGAGGTTTCCGACCATCACCTCCTCTTCTCGAATTTCACCACCAGTACCACCTTCGTCATCGAGAACTTGGAGGGCGGCACGGAGCTGGTCAAGGCGCGCCCCCGCCAGCTGGGCATGGTGATCCCGTTCGAGATGTCGCGCGTCCTGATCCCATCCGGCGCGAGCATCAGCGAACTCACCGTCTTCACTCCTCCGCCGCGCCTGCTCGGACCTGACCAGGCCGCCACGGTGGCTGAGTCCGCGATGACCAAGTTGGACCCGGAGAGCAAGTACTTCGCCGTTCTCGTCGCACTGTGCGAGCCGCGTCTGCGCGGTTCCTCCATGGCAGCCGTGCCCTCCGTTCAGGAGGTGGTCGAGAGGCTGAAGGGGATCAAGCAGTTCAGGGGTGCGAACCGCAGCAGCATCAACTACCACATCGACTACCTGACGGATCAGAAGCTTCCGGTCAGTCAGTGGGCCAAGTACGTGGACGAAGGCCGCATGCACTCCAAGCGCGAGGCCCTGGTCGCGTTCTCGCTCCGGTTCGACCTGGTGCGGGAGGAACACCTCGGTCTGCTCCCCGACCTCAAGCGTTCGAGGGCACAGCCTTCGTGAACTAGTTTTTTT |
| *aac(3)IV_*MRS-ISceI-cassette | AAAAAAATTCCGGGGATCCGTCGACCTAGGGATAACAGGGTAATACTAGTGTTTAAACCTTAAGCTGACGCCGTTGGATACACCAAGGAAAGTCTACACGAACCCTTTGGCAAAATCCTGTATATCGTGCGAAAAAGGATGGATATACCGAAAAAATCGCTATAATGACCCCGAAGCAGGGTTATGCAGCGGAAAATGCAGCTCACGGTAACTGATGCCGTATTTGCAGTACCAGCGTACGGCCCACAGAATGATGTCACGCTGAAAATGCCGGCCTTTGAATGGGTTCATGTGCAGCTCCATAAGCAAAAGGGGATG  ATAAGTTTATCACCACCGACTATTTGCAACAGTGCCGTTGATCGTGCTATGATCGACTGATGTCATCAGCGGTGGAGTGCAATGTCGTGCAATACGAATGGCGAAAAGCCGAGCTCATCGGTCAGCTTCTCAACCTTGGGGTTACCCCCGGCGGTGTGCTGCTGGTCCACAGCTCCTTCCGTAGCGTCCGGCCCCTCGAAGATGGGCCACTTGGACTGATCGAGGCCCTGCGTGCTGCGCTGGGTCCGGGAGGGACGCTCGTCATGCCCTCGTGGTCAGGTCTGGACGACGAGCCGTTCGATCCTGCCACGTCGCCCGTTACACCGGACCTTGGAGTTGTCTCTGACACATTCTGGCGCCTGCCAAATGTAAAGCGCAGCGCCCATCCATTTGCCTTTGCGGCAGCGGGGCCACAGGCAGAGCAGATCATCTCTGATCCATTGCCCCTGCCACCTCACTCGCCTGCAAGCCCGGTCGCCCGTGTCCATGAACTCGATGGGCAGGTACTTCTCCTCGGCGTGGGACACGATGCCAACACGACGCTGCATCTTGCCGAGTTGATGGCAAAGGTTCCCTATGGGGTGCCGAGACACTGCACCATTCTTCAGGATGGCAAGTTGGTACGCGTCGATTATCTCGAGAATGACCACTGCTGTGAGCGCTTTGCCTTGGCGGACAGGTGGCTCAAGGAGAAGAGCCTTCAGAAGGAAGGTCCAGTCGGTCATGCCTTTGCTCGGTTGATCCGCTCCCGCGACATTGTGGCGACAGCCCTGGGTCAACTGGGCCGAGATCCGTTGATCTTCCTGCATCCGCCAGAGGCGGGATGCGAAGAATGCGATGCCGCTCGCCAGTCGATTGGCTGAGCTCATAAGTTCCTATTCCGAAGCAGCTCCAGCCTACATTTTTT |

**References:**

1. Gust B, Challis GL, Fowler K, Kieser T, Chater KF. PCR-targeted *Streptomyces* gene replacement identifies a protein domain needed for biosynthesis of the sesquiterpene soil odor geosmin. Proc Natl Acad Sci USA. 2003;100:1541–6. https://doi.org/10.1073/pnas.0337542100

2. Grüschow S, Rackham EJ, Goss RJM. Diversity in natural product families is governed by more than enzyme promiscuity alone: establishing control of the pacidamycin portfolio. Chem Sci. 2011;2:2182. https://doi.org/10.1039/c1sc00378j

3. Erb A, Luzhetskyy A, Hardter U, Bechthold A. Cloning and Sequencing of the Biosynthetic Gene Cluster for Saquayamycin Z and Galtamycin B and the Elucidation of the Assembly of Their Saccharide Chains. ChemBioChem. 2009;10:1392–401. https://doi.org/10.1002/cbic.200900054

4. Flett F, Mersinias V, Smith CP. High efficiency intergeneric conjugal transfer of plasmid DNA from *Escherichia coli* to methyl DNA-restricting streptomycetes. FEMS Microbiol Lett. 1997;155:223–9. https://doi.org/10.1111/j.1574-6968.1997.tb13882.x

5. Paget MSB, Chamberlin L, Atrih A, Foster SJ, Buttner MJ. Evidence that the Extracytoplasmic Function Sigma Factor σE Is Required for Normal Cell Wall Structure in *Streptomyces coelicolor* A3(2). J Bacteriol. 1999;181: 204–11. https://doi.org/10.1128/JB.181.1.204-211.1999

6. Rackham EJ, Grüschow S, Ragab AE, Dickens S, Goss RJM. Pacidamycin biosynthesis: identification and heterologous expression of the first uridyl peptide antibiotic gene cluster. ChemBioChem. 2010;11:1700–9. https://doi.org/10.1002/cbic.201000200

7. Eustáquio AS, Gust B, Galm U, Li S-M, Chater KF, Heide L. Heterologous Expression of Novobiocin and Clorobiocin Biosynthetic Gene Clusters. Appl Environ Microbiol. 2005;71:2452–9. https://doi.org/10.1128/AEM.71.5.2452-2459.2005

8. MacNeil DJ, Km G, Cl R, G D, Ph G, T M. Analysis of *Streptomyces avermitilis* genes required for avermectin biosynthesis utilizing a novel integration vector. Gene. Gene; 1992;111:61–8. https://doi.org/10.1016/0378-1119(92)90603-m

9. Datsenko KA, Wanner BL. One-step inactivation of chromosomal genes in *Escherichia coli* K-12 using PCR products. Proc Natl Acad Sci USA. 2000;97:6640–5. https://doi.org/10.1073/pnas.120163297

10. Baba T, Ara T, Hasegawa M, Takai Y, Okumura Y, Baba M, et al. Construction of *Escherichia coli* K-12 in-frame, single-gene knockout mutants: the Keio collection. Mol Syst Biol. 2006;2:MSB4100050. https://doi.org/10.1038/msb4100050

11. Cherepanov PP, Wackernagel W. Gene disruption in *Escherichia coli*: TcR and KmR cassettes with the option of Flp-catalyzed excision of the antibiotic-resistance determinant. Gene. 1995;158:9–14. https://doi.org/10.1016/0378-1119(95)00193-a

12. Zhang W, Ostash B, Walsh CT. Identification of the biosynthetic gene cluster for the pacidamycin group of peptidyl nucleoside antibiotics. Proc Natl Acad Sci USA. 2010;107:16828–33. https://doi.org/10.1073/pnas.1011557107

13. Floriano B, Bibb M. *afsR* is a pleiotropic but conditionally required regulatory gene for antibiotic production in *Streptomyces coelicolor* A3(2). Mol Microbiol. 1996;21:385–96. https://doi.org/10.1046/j.1365-2958.1996.6491364.x

14. Gomez-Escribano JP, Bibb MJ. Engineering *Streptomyces coelicolor* for heterologous expression of secondary metabolite gene clusters. Microb Biotechnol. 2011;4:207–15. https://doi.org/10.1111/j.1751-7915.2010.00219.x

15. Hopwood DA, Kieser T, Wright HM, Bibb MJ. Plasmids, Recombination and Chromosome Mapping in *Streptomyces* *lividans* 66. J Gen Microbiol. 1983;129:2257–69. https://doi.org/10.1099/00221287-129-7-2257

16. Ehrlich J, Gottlieb D, Burkholder PR, Anderson LE, Pridham TG. *Streptomyces* *venezuelae*, N. Sp., the Source of Chloromycetin. J Bacteriol. 1948;56:467–77. https://doi.org/10.1128/jb.56.4.467-477.1948

17. Chater KF, Wilde LC. Restriction of a bacteriophage of *Streptomyces* *albus* G involving endonuclease SalI. J Bacteriol. 1976;128:644–50.

18. Myronovskyi M, Rosenkränzer B, Nadmid S, Pujic P, Normand P, Luzhetskyy A. Generation of a cluster-free *Streptomyces* *albus* chassis strains for improved heterologous expression of secondary metabolite clusters. Metab Eng. 2018;49:316–24. https://doi.org/10.1016/j.ymben.2018.09.004
